# Reversible solidification of fission yeast cytoplasm after prolonged nutrient starvation

**DOI:** 10.1242/jcs.231688

**Published:** 2019-11-01

**Authors:** Maria B. Heimlicher, Mirjam Bächler, Minghua Liu, Chieze Ibeneche-Nnewihe, Ernst-Ludwig Florin, Andreas Hoenger, Damian Brunner

**Affiliations:** 1 Department of Molecular Life Sciences, University of Zurich, Winterthurerstrasse 190, 8057 Zurich, Switzerland; 2 Dept. of Molecular, Cellular and Developmental Biology, University of Colorado at Boulder, UCB-0347, Boulder, CO 80309, USA; 3 Center for Nonlinear Dynamics and Department of Physics, University of Texas at Austin, Austin, TX 78712, USA

**Keywords:** Yeast, Cytoplasm immobilisation, Starvation, Autophagy

## Abstract

Cells depend on a highly ordered organisation of their content and must develop strategies to maintain the anisotropic distribution of organelles during periods of nutrient shortage. One of these strategies is to solidify the cytoplasm, which was observed in bacteria and yeast cells with acutely interrupted energy production. Here, we describe a different type of cytoplasm solidification fission yeast cells switch to, after having run out of nutrients during multiple days in culture. It provides the most profound reversible cytoplasmic solidification of yeast cells described to date. Our data exclude the previously proposed mechanisms for cytoplasm solidification in yeasts and suggest a mechanism that immobilises cellular components in a size-dependent manner. We provide experimental evidence that, in addition to time, cells use intrinsic nutrients and energy sources to reach this state. Such cytoplasmic solidification may provide a robust means to protect cellular architecture in dormant cells.

## Abstract (precis)

**Summary:** After prolonged quiescence, fission yeast cell populations switch state to immobilise subcellular components much more profoundly than cells experiencing acute energy depletion.

## INTRODUCTION

Cell function and survival require a highly ordered, cell type-specific organisation of the cytoplasmic content. How cells generate and maintain a cell type-specific spatial organisation is central to understanding the living state. It involves the asymmetric distribution of material, mediated by active transport of key regulatory components, which counteracts the entropic activity of diffusion. This occurs in a highly crowded and dynamic environment, with spatially dispersed constituents ranging in size from small ions and metabolites to macromolecular complexes, and to large, complex structures, like the cytoskeletal networks and organelles. Their sub-diffusive motion is influenced by macromolecular crowding, specific and unspecific interactions, and the polymer networks of the cytoskeleton (Luby-Phelps et al., 1986; Tolić-Nørrelykke et al., 2004; Weiss et al., 2004; Wirtz, 2009). Consequently, the cytoplasm has been described as a complex viscoelastic fluid, a gel-like material or a colloidal liquid at the transition to a glass-like state (Fels et al., 2009; Grygorczyk et al., 2015; Luby-Phelps et al., 1986; Miermont et al., 2013; Mitchison et al., 2008; Moeendarbary et al., 2013; Parry et al., 2014; Tolić-Nørrelykke et al., 2004). The maintenance of such a complex, anisotropic cellular architecture and its remodelling in response to environmental changes requires a constant input of energy. Organisms have adopted a variety of strategies to cope with situations in which energy is limited. For example, when nutrients become limiting for growing and dividing cells, they can exit the cell division cycle and enter quiescence, similar to when they start differentiating (Coller et al., 2006). Such nutrient starvation-induced quiescence reverses as soon as nutrients become available again (Lennon and Jones, 2011; Yanagida, 2009). In extreme cases of energy shortage, cells can enter a dormant state of little or no energy consumption (Lennon and Jones, 2011). This mainly involves the formation of specialised cell types, such as spores and seeds, some of which can survive for centuries. Some animal species, such as tardigrades or hibernating mammals, manage to adopt dormant states by massively downregulating their cellular metabolism (Guppy and Withers, 1999; Reuner et al., 2010; Storey and Storey, 2007). In most cases, however, it is unknown to which extent – if at all – the low energy consumption during such dormant states allows cells to maintain their favoured cytoplasmic organisation. One way to preserve cellular architecture during an extended dormancy period is to reduce the water content. This increases macromolecular crowding, which can trigger a transition into a glass-like state of the cytoplasm, constraining the motion of its cytoplasmic components. This mechanism has been shown to operate in bacterial spores, plant seeds, metabolically inactive bacteria and budding yeast cells acutely depleted of energy (Cowan et al., 2003; Dijksterhuis et al., 2007; Joyner et al., 2016; Munder et al., 2016; Parry et al., 2014; Sun and Leopold, 1997). Alternatively, cells replace water with high amounts of carbohydrates, possibly providing cytoplasmic vitrification to prevent harmful fusion events (Elbein et al., 2003; Soto et al., 1999). Furthermore, quiescent cells have been shown to introduce structural changes to cytoplasmic components following energy depletion, with many metabolic enzymes and other proteins forming transient assemblies (Laporte et al., 2008; Narayanaswamy et al., 2009; Noree et al., 2010; O'Connell et al., 2012; Petrovska et al., 2014; Sagot et al., 2006). These assemblies have been suggested to inactivate enzymatic function and to serve as storage depots. As a collective, they have been proposed to form higher-order structures that mediate a solid-like state of the cytoplasm (Munder et al., 2016).

When nutrients become growth limiting for cells of the fission yeast *Schizosaccharomyces pombe*, the cells will mate and produce dormant spores (Egel, 1989; Tanaka and Hirata, 1982). Cells that lack a mating partner will enter a quiescent state. So far, research on fission yeast starvation has largely been focused on the physiology of nitrogen-starved cells or on cells acutely depleted of glucose (Costello et al., 1986; Joyner et al., 2016; Oda et al., 2015; Saitoh and Yanagida, 2014; Yanagida et al., 2011). We describe fission yeast cells that have slowly run out of nutrients. After several days of culturing, these cells showed a sudden, drastic solidification of their cytoplasm, which we termed ‘cytoplasmic freezing’ (CF). In this state, structures with a diameter of 250–350 nm, show virtually no motion, while molecules the size of GFP (i.e. 4.5×2.5 nm) can almost freely diffuse. Cytoplasmic solidification of CF cells, is much more profound and robust than in cells with other known liquid- to solid-like transitions, and we show that a different molecular mechanism must be involved (Joyner et al., 2016; Munder et al., 2016).

## RESULTS

### Starved cells have two intracellular immobilisation states

**Fig. 1.**
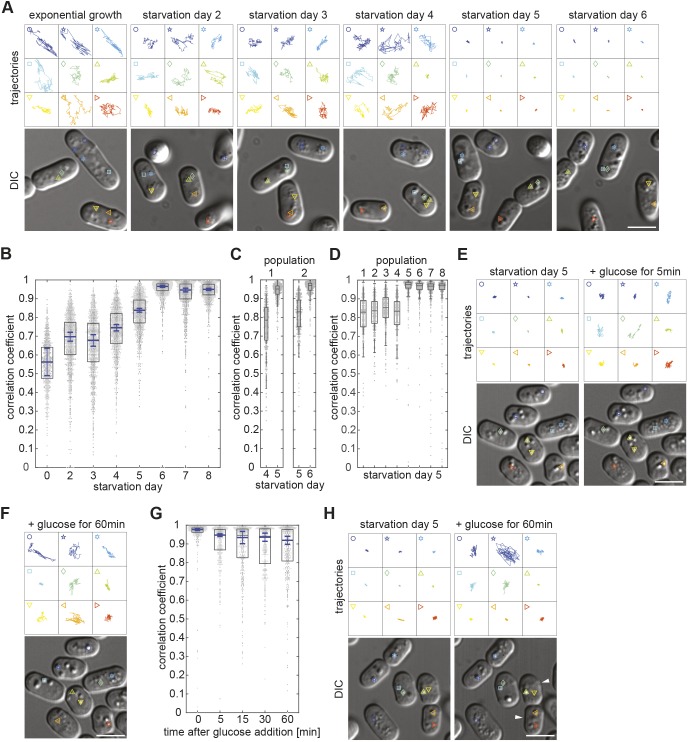
**Reversible motion arrest of LDs in deep starvation.** (A) Upper panels show trajectories of the LDs depicted in the DIC images (lower panels), with cells taken from 25-s movies (four frames) on different days of starvation. (B) Dot plots (1 dot/cell) with overlain box plots showing PCC quantification of BODIPY-labelled LD dynamics. Boxes represent the 25–75 percentile. The blue line shows the mean of the medians from four independent cell populations (*n*=820, 1451, 1406, 1380, 1407, 1308, 1260, 1241 cells). Error bars: 95% confidence interval. Note that none of the four cell populations had fully entered CF on SD5. Standard box plots are overlain. (C) Dot plot as in B, showing different populations and their timing of transition to CF (SD4 and 5, SD5 and 6; *n*=356 and 372, 325 and 357 cells, respectively). (D) Dot plot as in B, of different SD5 cell populations (*n*=323, 375, 387, 323, 453, 377, 424, 327 cells). (E) LD trajectories as in A, of cells on SD5, immediately before and 5 min after glucose addition. (F) LD trajectories as in A, of cells 60 min after glucose addition. (G) Dot plots as in B, showing LD dynamics at consecutive time points during starvation exit (three independent cell populations, *n*=354, 303, 442, 300, 400 cells). (H) LD trajectories as in A, of cells on SD5 immediately before and 60 min after glucose addition, showing LDs remaining immobilised in two of the cells (white arrowheads). All scale bars: 5 µm (A,E,F,H).

After 2 days in culture under standard conditions, fission yeast cells have used all the glucose in the medium and arrested growth to enter a quiescent state (Fig. S1A) (Makushok et al., 2016). Imaging cells during the following days, revealed a striking decrease in mobility of virtually all subcellular structures visible by light microscopy, ending with their seemingly complete immobilisation (Movie 1). An exception were small particles that erratically moved within vacuoles throughout starvation. We quantified this mobility change by tracking the motion of lipid droplets (LDs), which are clearly distinguishable in our differential interference contrast (DIC) microscopy images. We refer to 2 days in culture as starvation day 2 (SD2), 3 days in culture as starvation day 3 (SD3) etc. In exponentially growing cells the dispersed LDs dynamically move throughout the entire intracellular space (Fig. 1A; Movie 1). On SD2, LDs frequently accumulated into 1–2 grape-like structures that displayed visible motion, although their overall position within cells remained fairly constant (Fig. S1B). Between SD3 and SD4, LDs showed similar dynamicity but, increasingly, cells contained fewer, larger LDs, presumably due to fusion within the grape-like structures (Fig. S1B). Between SD5 and SD6, a larger proportion of cells also contained enlarged LDs in addition to the smaller ones. All these LDs, independently of their size, displayed complete absence of motion throughout the following days (Fig. 1A; Movie 1; Fig. S1C). We quantified this drastic mobility change, with a newly developed procedure for automated motion quantification of BODIPY-labelled LDs (Listenberger and Brown, 2007; Long et al., 2012). Simultaneous phloxine B treatment enabled automatic exclusion of dead cells from analysis (Fig. S1D; Materials and Methods) (Noda, 2008). Unfortunately, phototoxicity prevented the prolonged imaging required to calculate LD mean square displacement mobility. Instead, we determined a measure commonly used to quantify the colocalisation of two fluorescent markers, the Pearson correlation coefficient (PCC) (Adler and Parmryd, 2010; Dunn et al., 2011). We used the PCC to quantify the ‘colocalisation’ of a fluorescent marker with itself at two time-points separated by 42 s (Fig. S1E–G). The maximal PCC value of 1 describes a completely static distribution of fluorescence intensity and, thus, absence of particle displacement. Increased mobility of fluorescent structures decreases the PCC value accordingly. Notably, this method merely provides a measure of whether there is motion but not of its nature. By monitoring the values of all pixels of a cell between two consecutive time points, we obtained a PCC value of 0.56 in exponentially growing cells, reflecting the strong, seemingly erratic motion of LDs, including occasional jumps (Fig. 1B; Movie 1). After cells had entered a quiescent state at SD2, LD mobility was reduced and LDs mainly wobbled in a given location without major translocations (Movie 1). Accordingly, the PCC value increased to 0.70 (Fig. 1B). On SD3 there was little change, with average median PCC values of 0.68. On SD4, we measured a slight increase to a PCC value of 0.75. Only on SD5 did the average median PCC value start to increase significantly, i.e. to 0.84. On SD6, it reached 0.966, consistent with complete LD immobilisation (Fig. 1B). Thereafter, the average median PCC value remained high, i.e. 0.95 on SD7 and 0.95 on SD8. Remarkably, analysis of individual cell cultures showed that, in a given cell population, the final mobility reduction step was abrupt, occurring either on SD5 or SD6 (Fig. 1C). On SD5 the average median PCC value of a given culture remained either close to that measured for cultures on SD4, or jumped to that measured for all SD6 cultures (Fig. 1D). These results suggest that, within one population, cells transit to the state of cytoplasmic freezing in a coordinated fashion within a relatively short period of time*.* Occasionally, we noticed that an entire population of cultured cells did not transit to the CF state between SD5 and SD8 (Fig. S1H). Since such cell populations could be clearly distinguished, we excluded them from subsequent analysis. Altogether, our measurements suggested the presence of two consecutive starvation phases that are each characterised by an abrupt decrease of LD mobility. We termed these two intracellular immobilisation states ‘early starvation’ and ‘deep starvation’ and, the state of cells during deep starvation, where LDs are immobilised is, therefore, referred to as CF.

To investigate how cells regain LD mobility when exiting starvation, cells in the CF state were supplied with fresh growth medium (see Materials and Methods). LD mobility became detectable within <5 min, indicating that their release is triggered in a in a switch-like manner (Fig. 1E; Movie 2). LD mobility increased over the following hour, which led to broadening of the PCC value distribution (Fig. 1F,G). Some cells remained in the CF state during this observation period (Fig. 1H, white arrowheads; Movie 2).

### CF provides robust preservation of the cell shape

**Fig. 2.**
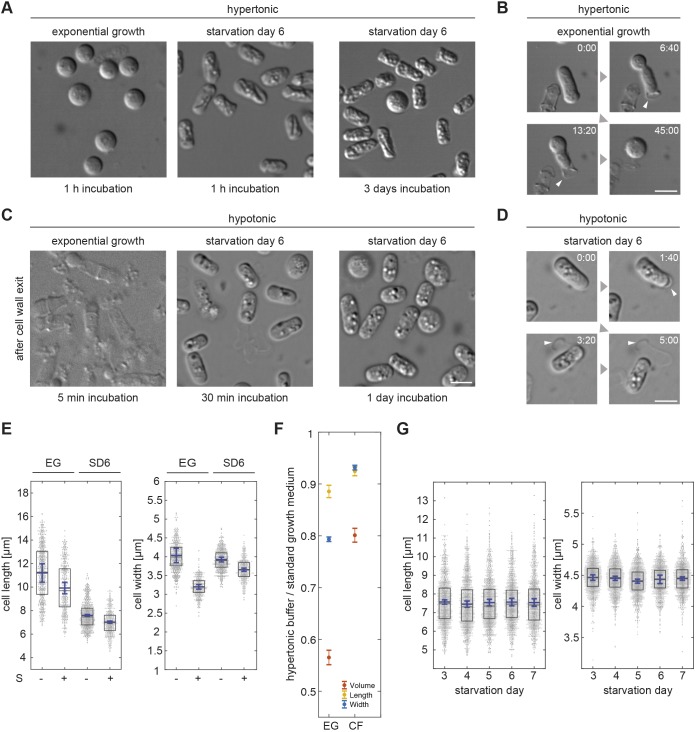
**Robust, cell wall-independent CF cell shape preservation without fluid loss.** (A) Protoplasts of cells incubated with cell wall-digesting enzymes in hypertonic buffer for 1 h (left panels) and for 3 days (right panel). (B) Live imaging of an exponentially growing cell and its protoplast exiting its cell wall (white arrowhead) when incubated with cell wall-digesting enzymes in hypertonic buffer. (C) Cell debris (exponentially growing cells) and protoplasts (CF cells) of cells incubated with cell wall-digesting enzymes in hypotonic buffer. (D) Live imaging of a CF cell and its protoplast exiting its cell wall (arrowheads) when incubated with cell wall-digesting enzymes in hypotonic buffer. (E) Distribution of cell length and width measured from DIC images of exponentially growing cells and CF cells in standard culture medium (−S) or incubated for 1 h in hypertonic buffer (+S) (three independent cell populations, *n*=547, 391, 643, 404 cells). Boxes represent the 25–75 percentile. The blue line shows the mean of three experimental means. Error bars: 95% confidence interval. (F) Ratios for cell lengths (yellow), widths (blue) or approximated volumes (red) comparing cells incubated for 1 h in hypertonic buffer to cells taken from standard growth medium (for details see Materials and Methods). Dots represent means; error bars: 95% confidence interval of bootstrapped mean ratios (see Materials and Methods). (G) Dot plots showing the cell length (left) and cell width (right) distributions from SD3 to SD7. Boxes represent the 25–75 percentile. Blue lines show the mean of three means extracted from three independent cell populations (*n*=1406, 1380, 1407, 1308, 1260 cells). Error bars: 95% confidence interval. All scale bars: 5 µm (A–D).

To further test the extent of cytoplasm solidification in CF cells, we generated protoplasts by digesting the cell wall. When applying the standard protocol for cell wall digestion, exponentially growing cells, as expected, adopted a spherical shape. By contrast, CF cells treated this way remained cylindrical. Since CF cells might have an altered cell wall composition making them resistant to digestion of the cell wall, we followed cell treatment with live imaging. This confirmed that, similar to exponentially growing cells, the majority of CF cells exited their cell wall shells. While exponentially growing cells squeezed through a small opening with massive deformation in order to immediately adopt a spherical shape, CF cells slipped out of their cell wall shells as solid cylinders (Movie 3). Consistently, this came with a delay of 2–4 min, as it required a larger opening in the cell wall. These data show that CF cells can preserve their shape independent of their cell wall (Fig. 2A). Shape preservation was robust as, even after an additional 3 days of incubation in the presence of the digestive enzymes, the majority of SD6 protoplasts remained cylindrical (Fig. 2A).

The standard protocol to create protoplasts uses a hypertonic digestion mix (1.2 M sorbitol), which strongly dehydrates exponentially growing cells to prevent them rupturing (Atilgan et al., 2015). Dehydration strongly increases macromolecular crowding, which could be crucial to preserve the cell shape of CF cells. To investigate this possibility, we removed the cell walls of CF cells by using a hypotonic digestion mix containing 0.5 M sorbitol only. Under this condition all exponentially growing cells rapidly lysed and no surviving protoplasts formed (Fig. 2C; Movie 4). By contrast, most CF cells still formed cylindrical protoplasts. Again, live imaging confirmed that the majority of CF cells exited their cell wall (Movie 4). Around 70% of the protoplasts remained cylindrical even after an additional incubation day (Fig. 2C). This indicates that dehydration-mediated macromolecular crowding is not responsible for CF protoplast shape preservation. We conclude that CF cells stably fix the entire cellular content in a way that makes them resistant to osmotic pressure.

CF protoplasts produced under hypotonic conditions had a smooth surface, whereas – under hypertonic conditions surfaces were shrivelled and indented, suggesting that CF cells can lose some fluid (compare Fig. 2A,C). To quantify the extent to which CF cells dehydrate in a hypertonic environment, we placed them in a buffer solution containing 1.2 M sorbitol and measured the changes in cell width – the best indicator of volume loss – and cell length. While exponentially growing cells shrank in width and length on average by 0.83 µm and 1.28 µm, respectively, CF cells only shrank by 0.27 µm and 0.58 µm, respectively (Fig. 2E). From these values we estimated cell volumes, assuming a cylinder plus two half spheres representing the cell ends. The predicted volume loss of 19.9% for CF cells was much less than the 43.5% of exponentially growing cells (Fig. 2F; see Materials and Methods). It is possible that CF cells are already severely dehydrated and, therefore, cannot shrink much further. Such dehydration could well be causative for CF. To test this possibility, we measured the changes in cell length and width, with daily measurements starting on SD3, which is when most cells had arrested growth and division, and until SD7. We found no change regarding cell lengths, and widths remained similar to those of freshly divided exponentially growing cells (Fig. 2G). Most importantly, no change occurred when cells entered the CF state at SD5 or SD6. This indicates that CF is not linked to fluid loss.

### Subcellular architecture during deep starvation

**Fig. 3.**
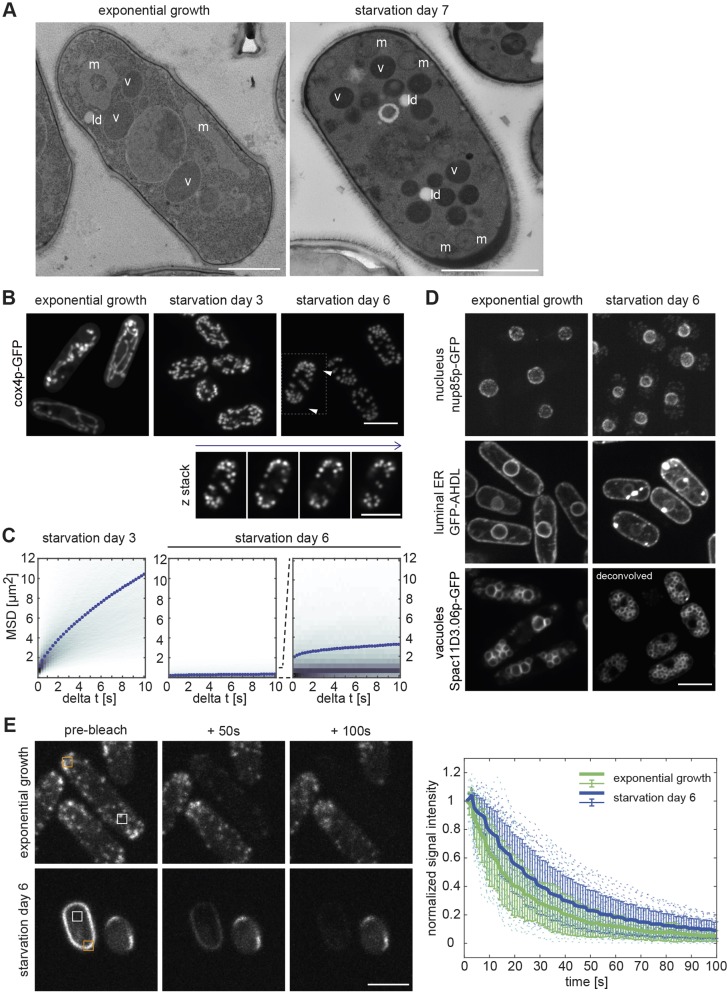
**Subcellular architecture in deep starvation.** (A) Electron micrographs of freeze-substituted, plastic-embedded and sectioned cells during exponential growth (left) and on SD7 (right), showing vacuoles (v), low-density lipid droplets (ld) and tube-like or spherical mitochondria (m; left or right panel, respectively). Note that sections do not show the cell centre. (B) Mitochondria visualised by maximum intensity projections of cox4p-GFP during starvation. The fragmented mitochondria on SD6 are often polarised (white arrowheads) and mostly cortical, as seen in the single planes of a *z*-stack (bottom panel; showing the boxed cell in the top panel). (C) Mean square displacement of spherical mitochondria at SD3 and SD6 (300 frames, four frames per second, six independent experiments, *n*=17,439, 15,948 particles; 15 particles per cell on average). Plotted are colour-coded histograms of the time-averaged mean square displacement of each particle. Dotted lines show the ensemble- and time-averaged mean square displacements. The second plot of SD6 (right panel) is a magnification of the first one (middle panel). Notice that for the first two panels *y*-axis numbers are ×10^−3^, in the last panel ×10^−4^. (D) Comparison of exponentially growing and SD6 cells, expressing markers of the nucleus (nup85p-GFP), vacuoles (Spac11D3.06p-GFP) or the ER (GFP-AHDL). The faint cytoplasmic dots in nup85p-GFP-expressing cells at SD6 represent autofluorescence rather than a nup85p-specific signal (see Fig. S2C). Images show single planes, deconvolved where indicated. (E) FLIP experiments using Lifeact-GFP in cells that were grown exponentially (upper panels) and in cells at SD6 (lower panels). Images show maximum-intensity projections within three planes. Repeated bleaching at the orange square was every 5 s. Plot shows the bleach-corrected and normalised mean signal intensity of the white squares. Thick lines indicate the mean of the normalised mean signal intensity of pooled cells from three independent experiments (*n*=27, 32 cells); error bars indicate the 95% confidence interval of the mean; dotted lines indicate the normalised signal of single cells. Scale bars: 1 µm (A), 5 µm (B,D,E).

To learn more about the cellular state of CF cells, we performed electron microscopy on plastic sections of SD7 cells. Superficially, the cells did not differ much from exponentially growing cells (Fig. 3A). Cell walls were similar in thickness and appearance. Organelles were surrounded by regions without any distinct structure, except for electron-dense spots typical of ribosomes. We found neither evidence for increased crowding of sub-cellular structures nor for any kind of filamentous assemblies. However, CF cells lacked the typical elongated mitochondria that span the length of exponentially growing cells (Fig. 3B). Instead, the mitochondria were small and globular with an approximate diameter of 250–350 nm (Fig. 3B). Most of these globular mitochondria were located in polar regions near the cell periphery (Fig. 3A,B). By contrast, organelles, such as LDs and vacuoles, had the tendency to cluster around the nucleus in the cell centre (Fig. 3A; Movie 1; Fig. S1B).

To further explore the sub-cellular architecture of CF cells, we analysed cells expressing GFP- or mCherry-tagged proteins, which are known to mark cellular organelles in exponentially growing cells (Table S1). When imaging control cells not expressing any markers with a 488 nm excitation laser and the GFP filter set at SD6, we noticed the gradual appearance of multiple autofluorescent particles (Fig. S2A). By SD6, this autofluorescence had reached such significant level that it had to be considered when imaging GFP-tagged proteins.

Comparing the known protein localisation of exponentially growing cells with that in cells on SD6, we found that GFP targeted to mitochondria via a fusion with precytochrome oxidase subunit IV (cox4p-GFP), significantly changed localisation (Fig. 3B) (Yaffe et al., 2003). In exponentially growing cells cox4p-GFP revealed the typical tubular mitochondria organisation. On SD3, mitochondrial tubes were shorter, and multiple dynamic and globular structures of uniform size became visible, suggesting that the mitochondria were undergoing fission (Fig. 3B; Movie 5). Consistently, on SD6 only globular mitochondria of uniform size were present, mainly near cell poles, as in our electron microscopy images (Fig. 3B). Similar to those in LDs, the motion of these globular mitochondria had completely arrested (Movie 5). Since cox4p-GFP fluorescence was sufficiently bright and stable, we could produce movie sequences that allowed us to determine the mean square displacement of mitochondria. This provided a second, independent means to quantify the CF state using a cellular component that is comparable in size with LDs but with a different subcellular location. On SD3, the mean square displacement of globular mitochondria was consistent with the significant, visible motion. On SD6 the mean square displacement was nearly zero, substantiating an almost complete immobilisation of organelle motion also in polar cell regions (Fig. 3C). This suggests that CF is a comprehensive phenomenon of the cytoplasm.

Unlike cox4p-GFP localisation of GFP-tagged nucleoporin nup85p did not change much in CF cells compared with that in exponentially growing cells (Fig. 3D). Similarly, GFP-tagged AHDL – a luminal marker of the nuclear envelope and the endoplasmic reticulum – still showed the alignment of ER with the entire cell membrane, nuclear envelope localisation and additional, ill-defined cytoplasmic membrane structures. Although additional bright dots appeared, this is indicative of an intact ER–membrane network, suggesting that no major changes in ER and nucleus organisation occur upon deep starvation. Vacuoles were also detectable at SD6, albeit at a clearly reduced size, while their number appeared to have increased (Fig. 3D). In addition, the vacuolar marker Spac11D3.06p-GFP confirmed that at SD6, vacuoles light up in all cells expressing mCherry-tagged proteins, suggesting vacuolar mCherry accumulation following degradation of the attached protein (Fig. S2B) (Costantini et al., 2015).

The signals of all other organelle markers vanished below the autofluorescence level (Fig. S2C). This suggests that, during deep starvation, many proteins are either downregulated or lose their distinct localisation, such that they no longer are detectable by fluorescence imaging.

### Immobilisation of cytoplasmic components in CF cells correlates with their size

Having shown that fragmented mitochondria and LDs – both ranging in size between ∼250 nm and 350 nm – are immobilised in CF cells, we wondered whether this also applies to components as small as individual proteins. To test this, we used cells expressing Lifeact-GFP (Huang et al., 2012; Riedl et al., 2008), a globular 29 kDa protein of 4.5×2.5 nm, which binds to F-actin with a high turnover rate and otherwise seems biochemically inert. It makes it a good readout compared to endogenous proteins, which are more likely to be subject to unknown transient binding interactions (Riedl et al., 2008; Yang et al., 1996). To test the mobility of the free, cytoplasmic Lifeact-GFP pool, we used the fluorescence loss in photobleaching (FLIP) method (Bancaud et al., 2010). FLIP involves repetitive bleaching of a defined, cellular sub-region. The kinetics of fluorescence loss in the remaining, unbleached cell regions provide a good measure of fluorescent particle motion into the bleached regions, thereby revealing information about the diffusive behaviour of the particle. In CF cells on SD6, Lifeact-GFP depleted fast into unbleached regions (Fig. 3E). Notably, the strong signal on the stable actin cables also depleted fast. This revealed fast binding and unbinding between the fluorescent protein and actin, as well as a rapid diffusion of Lifact-GFP throughout the cytoplasm that is not significantly reduced when compared to its diffusion rate in exponentially growing cells (Fig. 3E). We concluded that, in CF cells, larger cellular components are fixed in place, whereas small molecules can diffuse almost as freely in CF cells as in exponentially growing cells. This is consistent with the presence of a global network structure comprising a certain mesh size in CF cells.

### CF is not mediated through the cytoskeleton

To exclude fluid loss, we next investigated the possibility that cytoskeletal filaments form a cell-wide meshwork to trap bigger structures, such as LDs. Microtubules can be virtually excluded, as previous work has shown that they completely disappear from cells at on about SD4–SD5, with the exception of a single, very short microtubule stump remaining in some of the cells (Laporte et al., 2015; Makushok et al., 2016). We confirmed this result by imaging cells that express GFP-tagged alpha2 tubulin (Fig. S3A). In addition, we show that, in CF cells at SD6, no microtubules reformed.

**Fig. 4.**
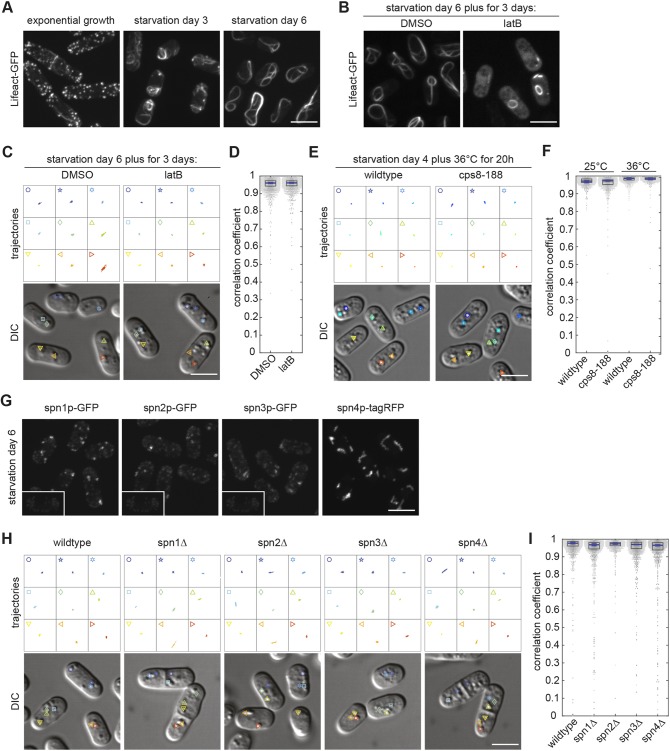
**Interference with cytoskeleton does not affect CF.** (A) Lifeact-GFP visualising F-actin during starvation. (B) Lifeact-GFP from cells on SD6 that were incubated with LatB or DMSO from SD3 onwards. (C) LD trajectories extracted from 25-s movies (four frames per second, droplets depicted in lower DIC images) of wild-type cells on SD6 incubated with DMSO or LatB from SD3 onwards. (D) Dot plots (one dot per cell) showing PCC-based quantification of BODIPY-labelled LD dynamics of wild-type cells on SD6 from three independent cell populations incubated with DMSO or LatB from SD3 onwards. Boxes represent the 25–75 percentile. The blue line represents the mean of the three medians extracted from three independent cell populations (*n*=1539, 1148 cells). Error bars show the 95% confidence interval. (E) LD trajectories (as described in C) of wild-type cells and cells carrying the temperature-sensitive actin mutation *cps8-188* at SD5. At SD4, temperature was increased from 25°C to 36°C for 20 h. (F) Dot plots as described in D, showing quantification of LD dynamics of wild-type and *cps8-188* cells at SD5 and SD6, respectively, at 25°C (left), and after a shift in temperature at SD4 from 25°C to 36°C for 20 h (right, as in E) (*n*=1056, 1059, 956, 907 cells). (G) Localisation of GFP-tagged spn1p, spn2p, spn3p and tagRFP-tagged spn4p on SD6. The unspecific signal portion can be estimated by comparing the autofluorescence from a SD6 wild-type cell without a fluorescent protein tag having used the same imaging and contrast settings (insets). (H) LD trajectories as in C, of wild-type cells and cells expressing deletion mutant *spn1*Δ, *spn2*Δ, *spn3*Δ or *spn4*Δ on SD6. (I) Dot plot representation as in D, showing quantification of LD dynamics in wild-type cells and cells expressing the deletion mutants *spn1*Δ, *spn2*Δ, *spn3*Δ and *spn4*Δ on SD6 (*n*=1529, 1311, 1062, 1434, 1044 cells). Fluorescence images represent maximum intensity projections. All scale bars: 5 µm.

To test for a role of F-actin in CF, we again used cells expressing the F-actin marker Lifeact-GFP (Huang et al., 2012; Riedl et al., 2008). As previously shown, in exponentially growing cells Lifeact-GFP labelled thin actin filament bundles that align parallel to the long cell axis and dynamic actin particles that concentrated at growing cell poles (Huang et al., 2012). On SD2–SD4, the actin particles disappeared, except for a few remaining dynamic patches that were distributed throughout the cells. The thin interphase filaments were replaced by thicker, dynamic F-actin bundles (Fig. 4A; Movie 6). These extended along the cell periphery and often curled around the cell ends or curled up inside the cells. On SD6, all actin patches had disappeared and the F-actin bundles had evolved into extremely prominent, very long F-actin bands (Fig. 4A). These bands were completely immobile and extended along the cell circumference, while curling around both cell ends to form a structure often reminiscent of a shoelace (Fig. 4A; Movie 6). The very strong signal of these actin bands suggests that they contain most of the cellular actin pool. Nevertheless, this does not fully rule out that the cells additionally harbour a global F-actin network. Unlike single microtubules, single actin filaments cannot be detected by fluorescence imaging or standard electron microscopy. We, therefore, treated cells with latrunculin B (LatB), a toxin that interferes with F-actin polymerisation (Spector et al., 1983). Addition of LatB to exponentially growing cells resulted in fast depletion of all visible F-actin structures (Fig. S3B). By contrast, addition of LatB to cells on SD6 did not affect the shoelace-like F-actin structure, suggesting that these do not turn over – unless the drug cannot enter CF cells (Fig. S3C). As this result did not allow to draw conclusions on the role of F-actin, we applied LatB prior to induction of CF (i.e. on SD3) and incubated cells in the continued presence of the drug until SD6 (see Materials and Methods). This prevented the formation of the shoelace-like F-actin structures in many cells, so they ended up with a dispersed Lifeact-GFP signal (Fig. 4B). In the other cells, the marker labelled short stumps and ring-like F-actin structures. As, in this experiment, LatB significantly interferes with the formation of thick F-actin cables in CF cells, it is unlikely that it does not do so with a network of single actin filaments. Nevertheless, such cells showed CF on SD6 – which is similar to DMSO-treated control cells (Fig. 4C,D; Movie 7). Similarly, *cps8-188* cells carrying a temperature-sensitive mutation in the actin-encoding *act1* gene, switched to the CF state at SD5 when shifted to the restrictive temperature at SD4 (Fig. 4E,F) (Ishiguro and Yamada, 1993). Together, these results suggest that F-actin does not crucially contribute to CF.

Septins are another protein family that has been shown to assemble into filaments that potentially form a global network in CF cells (Mostowy and Cossart, 2012). The fission yeast genome has seven non-essential septin genes (*spn1* to *spn7*) (Longtine et al., 1996; Pan et al., 2007). Of these, spn1p, spn2p, spn3p and spn4p assemble in the cytokinetic ring during cell division, whereas spn5p, spn6p and spn7p are exclusively expressed during meiosis (Fig. S3D) (Abe and Shimoda, 2000; Mata et al., 2002; Onishi et al., 2010; Watanabe et al., 2001). To ensure that the latter are not additionally expressed during deep starvation, we checked the signal of cells expressing GFP-tagged endogenous spn5p, spn6p and spn7p on SD6. We found no signal intensity above that of autofluorescence (Fig. S3E) and, therefore, carried on to analyse the non-meiotic septins spn1p to spn4p fused with GFP or tagRFP. We found that they all formed small clusters during deep starvation (Fig. 4G). Next, we tested cells carrying a deletion of any of the four septin genes *spn1* to *spn4*, which prevent septin filament formation and/or the correct formation of the septin ring in cytokinesis (An et al., 2004; Berlin et al., 2003; Tasto et al., 2003). On SD6, all cells expressing septin deletion mutants showed a CF that was indistinguishable from that in wild type cells (Fig. 4H,I). We, therefore, conclude that septins are unlikely candidates to mediate CF.

### Autophagy accelerates the establishment of CF

**Fig. 5.**
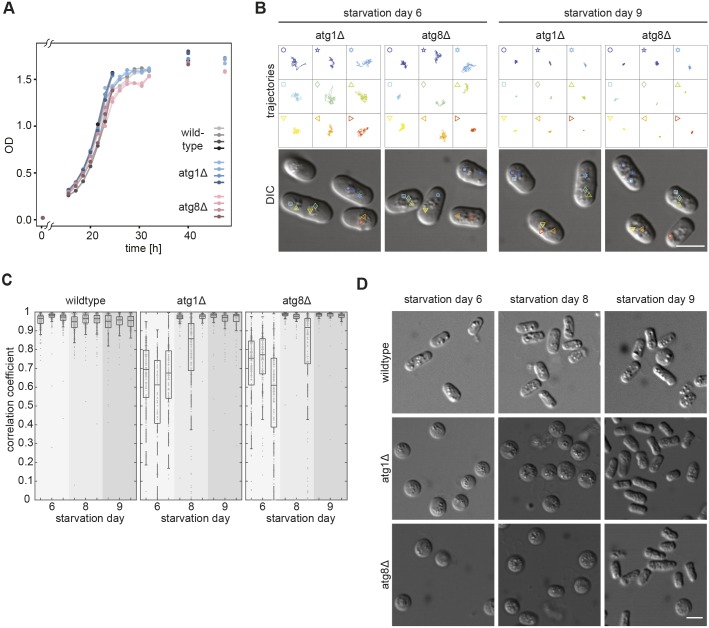
**Autophagy mutants delay transition to CF state.** (A) Optical density (OD) measurement-based growth curves of wild-type (grey), *atg1*Δ (blue) and *atg8*Δ (rose) cells (four independent cell populations each). (B) LD trajectories extracted from 25-s movies (four frames per second, droplets depicted in lower DIC images) of *atg1*Δ and *atg8*Δ cells on starvation days SD6 and SD9. (C) Dot plots (one dot per cell) showing PCC-based quantification of BODIPY-labelled LD dynamics for wild-type, *atg1*Δ and *atg8*Δ cells (three independent cell populations, *n*=657, 733, 481, 422, 521, 361, 598, 447, 296 cells). Boxes indicate the 25–75 percentile. (D) Protoplasts of wild-type, *atg1*Δ and *atg8*Δ cells at SD6 (left), SD8 (middle) and SD9 (right). Cell wall digestion for 4 h under conditions of 0.5 M sorbitol. All scale bars: 5 µm.

To further investigate the molecular nature of CF, we used our automated quantification of LD motion to screen for gene deletions that prevent CF in cells during deep starvation, by using a library of 3400 fission yeast strains that each carry a deletion of a non-essential gene (see Materials and Methods; Kim et al., 2010). The strains were screened on SD8 because, due to the presence of multiple auxotrophic mutations, these strains entered deep starvation with a delay. Of the roughly 500 deletions strains that did not show CF during deep starvation, we noticed a clear accumulation of mutants affecting autophagy. Autophagy is an evolutionarily conserved mechanism used by cells to remove damaged organelles and to recycle cellular components (Nakatogawa et al., 2009). To investigate the role of autophagy in CF, we took a prolonged look at two strains carrying a deletion of either *atg1* (*atg1*Δ) or *atg8* (*atg8*Δ), genes that both encode essential autophagy pathway components. Cells of both mutant strains showed normal exponential growth and entered quiescence at a time similar to that of wild-type cells (Fig. 5A), and both mutant strains entered the CF state, although with a 2–3-day delay compared with wild type (Fig. 5B,C; Movie 8). Consistently, cell wall digestion of *atg1*Δ and *atg8*Δ mutant cells in a hypotonic environment produced spherical protoplasts between SD6 and SD8. However, only at SD9 did they robustly maintain a cylindrical shape similar to that in wild-type cells at SD6 (Fig. 5D). These results indicate that autophagy is not essential for CF but that it promotes the ability of cells to enter the CF state.

### CF differs from other solid-like cytoplasmic states

**Fig. 6.**
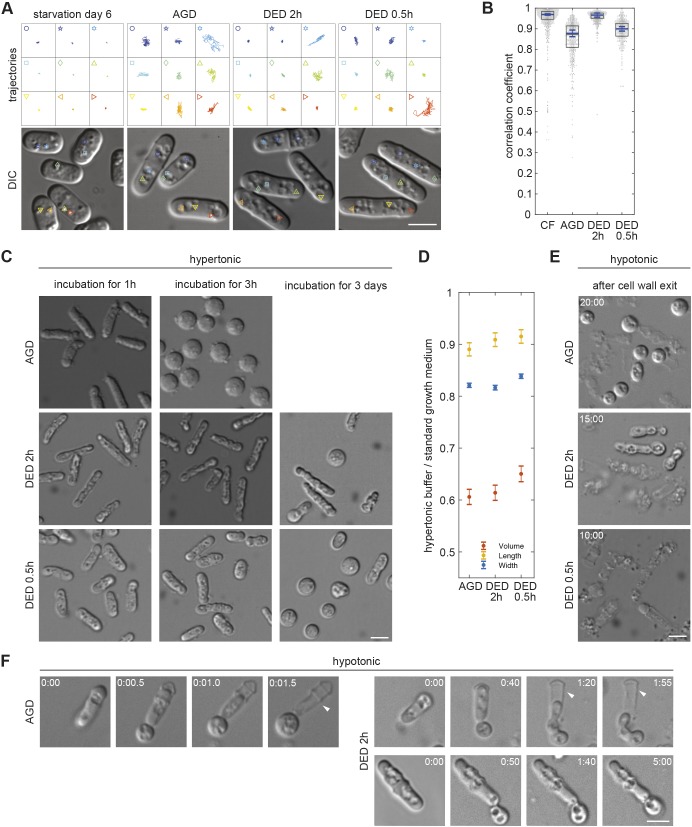
**CF differs from other solid-like cytoplasmic states.** (A) LD trajectories extracted from 25-s movies (four frames per second, droplets, depicted in the four DIC images shown) of CF cells on SD6, AGD cells and DED cells either 2 h or 0.5 h after drug exposure. (B) Dot plots (one dot per cell) showing PCC-based quantification of BODIPY-labelled LD dynamics in CF cells at SD6, in AGD cells and in DED cells with 2 h or 0.5 h of drug exposure. Boxes represent the 25–75 percentile. The blue line represents the mean of the three medians extracted from three independent cell populations (*n*=795, 562, 350, 447 cells). Error bars show the 95% confidence interval. (C) Protoplasts of cells incubated with cell wall-digesting enzymes in hypertonic buffer containing 1.2 M sorbitol for 1 h (left panels), 3 h (middle panels) or 3 days (right panels). (D) Ratios calculated from measured length (yellow), width (blue) or approximated volume (red) of cells placed in the respective standard culture medium, or in 1.2 M sorbitol containing buffer for 1 h. To estimate the variance of the three experiments, bootstrapping was performed (see Materials and Methods). Dots represent means, error bars show the 95% confidence interval of the bootstrapped mean ratios estimated with Gaussian error propagation. (E) Protoplasts or cell remnants from cells incubated with cell wall-digesting enzymes in hypotonic buffer containing 0.5 M sorbitol until complete cell wall exit (20, 15, 10 min). (F) Live imaging of protoplast evasion from the cell wall (white arrowheads) in the presence of 0.5 M sorbitol. Left: AGD cell, total time 1.5 s. Right: DED 2 h cells, total time <2 min (top) and 5 min (bottom). All scale bars: 5 µm.

Two recent studies reported on cytoplasm solidification in budding yeast cells, depleted of energy either by acute glucose depletion (AGD) or drug-induced energy depletion (DED) (Joyner et al., 2016; Munder et al., 2016). These states being reminiscent of CF, we directly compared the respective cells with cells during deep starvation (see Materials and Methods). For cells depleted of energy in response to DED (hereafter referred to as DED cells), drugs were applied for 0.5 or 2 h, as we found incubation times to be crucial (see Materials and Methods). Time-lapse imaging using DIC microscopy showed that, unlike in CF cells, LD motion was evident in cells depleted of energy in response to AGD (hereafter referred to as AGD cells) and in DED cells (Fig. 6A; Movie 9). Quantification with the BODIPY-labelling approach revealed an average median PCC value of 0.878 for AGD cells and of 0.900 for DED cells when cells were treated for 0.5 h (Fig. 6B). The average median PCC value – i.e. 0.966 – for DED cells treated for 2 h was similar to that of CF cells (0.969) but, unlike LDs in CF cells, some of their LDs clearly showed movement. Notably, the weaker BODIPY fluorescence in CF cells reduced the precision of automated analysis and resulted in a PCC value that was lower than in AGD and DED cells.

Both budding yeast studies contained a fission yeast experiment in which protoplasts were produced from AGD or 2 h-treated DED cells, using the standard protocol. These protoplasts were found to preserve their cylindrical shape unlike untreated cells that became spherical (Fig. 6C; Movie 10; see Materials and Methods) (Joyner et al., 2016; Munder et al., 2016). To investigate the robustness of shape preservation, we extended the observation period of protoplasts in the continued presence of digestive enzymes as done for CF cells (see Materials and Methods). We found that, within 3 h, the vast majority of the, initially, cylindrical AGD protoplasts had become spherical, indicating that these cells can still rearrange their cytoplasm, although with kinetics lower than that in exponentially growing cells (Fig. 6C). DED cells, by contrast, mostly remained cylindrical for the first 3 h. Only after 3 days of incubation ∼20% of the 2 h-treated and ∼80% of the 0.5 h-treated DED cells had turned spherical (Fig. 6C). This suggests that, under hypertonic conditions, robust cytoplasmic solidification can only be reached by prolonged drug exposure.

As mentioned above, the standard hypertonic cell wall digestion mix dehydrates cells to an extent that is crucial to preserve protoplast shape. Indeed, measurements of cell width and length showed that, when kept in a 1.2 M sorbitol solution, AGD and DED cells shortened, and considerably reduced their width (Fig. 6D; Fig. S4). Volume loss was estimated to be 39.4% for AGD cells, 35.1% for 0.5 h-treated DED cells and 38.6% for 2 h-treated DED cells (Fig. 6D). This shows that, under hypertonic conditions, AGD and DED cells experience increased macromolecular crowding due to considerable fluid loss within their cytoplasm. To investigate the contribution of volume loss to the preservation of protoplast shape, we produced protoplasts under hypotonic conditions (see Materials and Methods). As with exponentially growing cells, this caused rapid lysis of all DED cells treated for 0.5 h and in the majority of DED cells treated for 2 h (Fig. 6E; Movies 4 and 10). Time-lapse imaging revealed that the ∼15% of 2 h-treated cylindrical DED protoplasts did not fully exit the cell wall shell. The parts that did rounded up, suggesting a fluid content (Fig. 6F; Movie 10). Of the AGD cells, ∼50% lysed, while the remaining cells adopted a spherical shape, squeezing out of the cell wall shell in a manner that is similar to exponentially growing cells in hypertonic digestion solution (Fig. 6E,F; Movies 4 and 10). These results show that, unlike for CF cells, experimentally imposed dehydration is crucial for protoplast shape preservation of AGD and DED cells.

Taken together, these results indicate that the substantially more-profound cytoplasmic solidification of CF cells is different compared with that of cells experiencing acute energy depletion.

### Starved fission yeast cells lack extensive protein assemblies

**Fig. 7.**
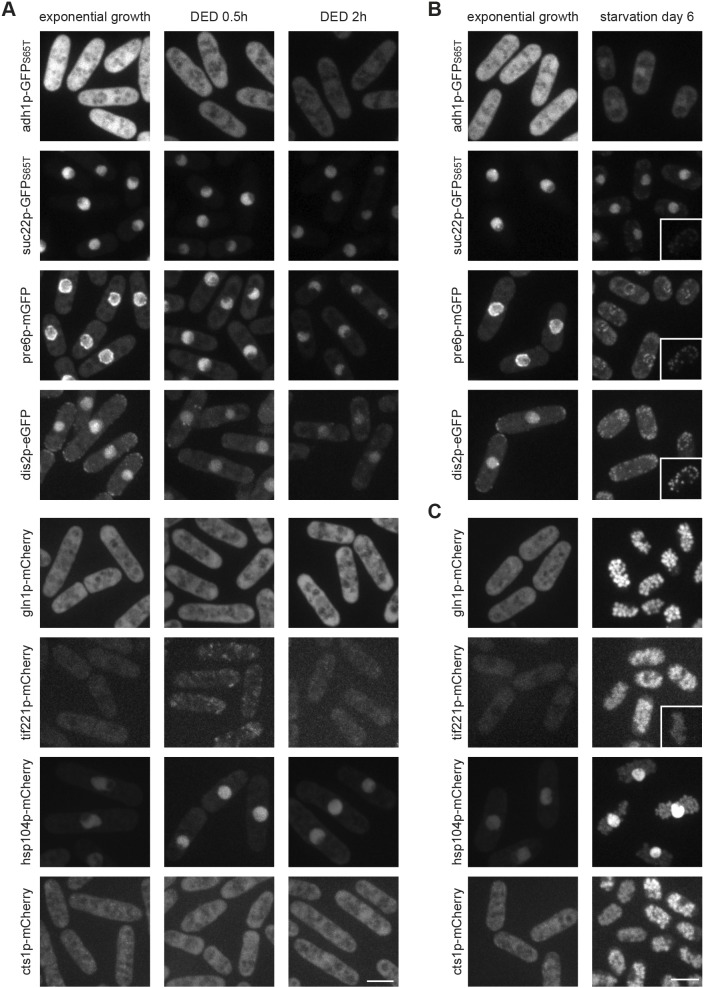
**Fission yeast cells do not form** **macromolecular protein assemblies.** (A) Images show fluorescence signal of the indicated fusion proteins in exponentially growing cells (left panels) and DED cells (right panels) incubated for 0.5 or 2 h prior to imaging. (B,C) Images show fluorescence signal of the indicated fusion proteins in exponentially growing cells (left panels) and SD6 cells (right panels). The unspecific signal portion can be estimated from comparison to the autofluorescence from an SD6 wild-type cell without a fluorescent protein tag with the same imaging and contrast settings (insets). Images are maximum intensity projections in all panels. All observations were confirmed in two independent experiments. All scale bars: 5 µm.

Cytoplasmic solidification of budding yeast DED cells was proposed to be mediated by the homotypic assembly of a large number of proteins (Joyner et al., 2016; Munder et al., 2016). To check for a similar behaviour of homologous proteins, we followed several of them, fused with mGFP and/or mCherry, in 0.5 h-treated and 2 h-treated fission yeast DED cells (Table S2). None of these proteins formed assemblies, suggesting that fission- and budding yeast cells react differently to DED (Fig. 7A). Importantly, when fused with GFP(S65T) instead of monomeric fluorescent protein tags, some of the tested proteins formed either DED-specific assemblies or assemblies in untreated as well as DED-treated cells (Fig. S5A).

Although our data do not support the formation of protein assemblies in DED fission yeast cells, we cannot exclude this possibility in CF cells. Therefore, we checked the same proteins in CF cells on SD6 and found no detectable aggregates. Moreover, signal intensities of most of GFP-tagged proteins had dropped below the aforementioned autofluorescence (Fig. 7B,C; Fig. S2A). Western blot analysis showed reduced protein levels for most of the tested proteins (Fig. S6). Notably, actin levels were also reduced further, supporting our above conclusion that CF is not mediated by F-actin. Also, pre-existing GFP(S65T)-dependent aggregates disappeared (Fig. S5B). As shown above, cells expressing mCherry-tagged proteins, showed fluorescence accumulation within the vacuolar lumen, in one case accompanied with bright nuclear fluorescence (Fig. 7C). Consistent with the absence of macromolecular assemblies in CF cells, electron microscopy pictures showed no evidence of distinct areas, as described for protein aggregates in budding yeast cells (Fig. 3A) (Petrovska et al., 2014). These results suggest that extensive macromolecular protein assemblies are unlikely to be the basic cause of CF.

## DISCUSSION

We, here, introduce CF as the strongest cytoplasmic solidification state of yeast cells found to date. CF occurs switch-like, after several days of quiescence only, in almost all cells of a glucose-starved population. This phenotype is not modulated by the stage of the cell cycle or the cell size because glucose-starved cells, with few exceptions, are of similar length and are mainly arrested in G2 – the stage of the cell cycle in which fission yeast cells mainly grow (Costello et al., 1986). CF significantly differs in many ways from the two previously described cytoplasmic solidification states that can be induced by either AGD or DED of exponentially growing cells (Joyner et al., 2016; Munder et al., 2016). Following acute energy depletion in fission yeast cells, LD motion is, indeed, much reduced but, in contrast to CF cells, still evident by eye. We quantitatively confirmed this difference with our cross-correlation analysis, which faithfully monitors LD displacement between two time points, thus, enabling direct comparison of LD motion between the different states with high sensitivity. Unfortunately, a more-standard comparison using LD mean square displacement was impossible for technical reasons. Such value could only be obtained for globular mitochondria in CF cells. The fact that these mitochondria have a diameter that is very similar to that of LDs and are found in different locations, suggests that, generally, motion of organelles in the 250 nm size range is virtually abrogated in CF cells. In addition to the differences regarding LD immobilisation, we found several other fundamental differences, indicating that the mechanism underlying CF differs from that with which exponentially growing cells respond to acute energy depletion (Joyner et al., 2016; Munder et al., 2016). First of all, CF cells very robustly preserve their cylindrical shape in the absence of a cell wall, even in a hypotonic solution, in which exponentially growing cells, as well as AGD and DED cells, rapidly lyse and disperse their cellular content. The previously published shape preservation of cell wall-depleted fission yeast cells under AGD and DED conditions does – we believe – depend on experimentally generated cellular dehydration, which critically increases sub-cellular, macromolecular crowding. In addition, under most conditions, this shape preservation was not robust and protoplast rounding occurred with a delay. Increased macromolecular crowding due to fluid loss has been proposed to underlie cytoplasmic solidification of AGD cells and to contribute in DED cells. Since we found no evidence for dehydration when quiescent cells switch to CF between SD5 and SD6, this mechanism cannot be critical. Consistently, we also find no evidence for dehydration during the days preceding CF induction, suggesting that CF is not just a more-profound or -advanced manifestation of the state induced by AGD. In DED cells, a pH-dependent formation of numerous homotypic protein assemblies has been proposed to be the other, more-crucial mechanism providing cytoplasmic solidification (Munder et al., 2016). In CF cells, we found no evidence for the presence of such assemblies, again arguing for a different mechanism underlying CF. So far, such homotypic protein assemblies have only been shown in cells of budding yeast. Interestingly, we did not find any evidence for such assemblies forming in similarly drug-treated fission yeast cells. Notably, this was true only when homologs of the relevant proteins had been fused with monomeric fluorescent protein tags. When fused with the GFP(S65T) variant – which is routinely used in budding and fission yeast research – some of these proteins, indeed, formed such assemblies (Huh et al., 2003). That certain fluorescent protein tags mediate protein aggregation and, thus, should be avoided when studying protein assemblies has previously also shown for bacterial cells (Landgraf et al., 2012). However, since we only analysed a limited number of proteins, we cannot exclude that, simply by chance, we might have missed the crucial ones.

Therefore, at this stage, we cannot specify the mechanism causing CF but it seems that quiescent cells need 3–4 days to become competent to switching to this state. In support of this, we found that when blocking autophagy, a conserved mechanism for cell autonomous recycling of macromolecules, the onset of CF is delayed by 2–3 days. It is plausible that cells use autophagy to provide energy and, possibly, to produce components that mediate the CF state in the absence of external sources. One possible mechanism is the specific enrichment of small components, such as sugars or other metabolites, in the cytosol. This strategy is used by animals to control the formation of subcellular ice crystals when exposed to temperatures below 0°C and by plants to increase the longevity of their seeds (Leprince et al., 2017; Storey and Storey, 2017). What speaks against such a mechanism in CF cells is that we would expect it to also decrease the motility of protein-sized components, such as Lifeact-GFP, which is not the case. Thus, another mechanism seems more plausible, in which CF cells generate a global polymer network that forms a stable mesh throughout the cytosol. Such a network would preserve the cell shape and fix larger cellular components, while allowing molecules smaller than the mesh size to freely move – as observed for Lifeact-GFP. Also in support of a polymer is the fact that, in a hypertonic environment, the fluid loss of CF cells is small as compared to exponentially growing cells or AGD and DED cells, given that the ability to retain fluid is typical for hydrogels. Alternatively, fluid retention in CF cells could be due to complete insulation of quiescent cells from the environment. Arguing against is the fact that the latter still take up BODIPY dye and still actively eliminate phloxine B, our marker to detect dead cells that fill up with the dye (Fullerton et al., 2006; Tamai et al., 1996). Notably, hydrogels can form spontaneously through phase transition once the crucial components have reached a certain concentration. This has been proposed to occur during various processes, including the formation of Balbiani bodies, and can involve proteins and/or RNAs (Boke et al., 2016; Brangwynne et al., 2009; Han et al., 2012; Jain and Vale, 2017; Kato et al., 2012; Patel et al., 2015). In our case, such phase transition might account for the tremendous synchrony with which CF occurs in a given cell population on either SD5 or SD6, assuming that all cells of a population produce the crucial components at a similar rate. Prime candidates that might form a polymer network are the known filament-forming proteins actin, tubulin or septins. However, protein localisation, electron microscopy imaging and interference experiments do not support a role for any of them.

It seems obvious that CF preserves overall cell architecture, thus, preventing cells to use vast amounts of energy in times of prolonged nutrient starvation. Also, CF may provide resistance to various stresses, without the need for metabolic activity. Unfortunately, our starvation conditions, which rely on standard fission yeast liquid culturing, are not optimal to investigate the role of CF in cell survival. The problem is that mortality rates massively increase after SD8. This is probably due to the fact that, under these conditions, cells enter starvation in the presence of their own toxic waste. Thus, in order to perform such studies, more favourable starvation conditions need to be established.

Nevertheless, CF is very much reminiscent of a recently published process in tardigrades, which is essential to provide tolerance to desiccation (Boothby et al., 2017). For this, the tardigrade cells first need some time to express intrinsically disordered proteins. These, eventually, will undergo a global phase transition that converts the cytoplasm into a gel-like state in which the cellular content is fixed (Boothby et al., 2017). Thus, key to understanding the mechanism that underlies CF is the identification of the responsible components. In addition, corresponding gene-deletion strains will allow testing whether the CF state is cytoprotective during a starvation period.

## MATERIALS AND METHODS

### Yeast cell culturing

Cells were grown at 25°C in Edinburgh minimal medium with 2% glucose (EMM2), supplemented with thiamine as required, and as described by Moreno et al. (1991). For CF experiments, cells were cultured to enter starvation as described by Zaitsevskaya-Carter and Cooper (1997). Standard pre-cultures in mid-exponential growth phase cultured in EMM2 were diluted to an OD of 0.02 (spectrophotometer Genesys 10S Vis, Thermo Fisher Scientific, Waltham, MA) in Edinburgh minimal medium with low (0.5%) glucose (EMMLG) plus supplements as required. Notably, CF occurred also when cells were diluted in EMM2 (standard starting condition to analyse exponentially growing cells) but the synchrony of induction was reduced and quantitative analysis was hindered by optical inhomogeneity amongst the cells.

Cells were cultured in Erlenmeyer flasks on a shaker (New Brunswick scientific innova 4230; 220 rpm) for ≤8 days. The culture volume did not exceed 1/10 of the total volume of the flask. All strains used are listed in Table S1.

To follow starvation exit, we added glucose by supplementing cells with fresh EMM2 (1:4) and incubating them in an Erlenmeyer flask on the shaker at 25°C. AGD was done as described for budding yeast (Joyner et al., 2016). DED was done as described (Munder et al., 2016), with antimycin A from *Streptomyces sp*. (A8674) 2-deoxy-D-glucose (D8375) all from Merck (Darmstadt, Germany). We performed all DED experiments using drug incubation for 0.5 or 2 h, as different incubation times have previously been reported for different experiments and for the different drugs tested (Munder et al., 2016). Glucose levels in filtered culture medium (Filtropur S 0.2, Sarstedt 7510401) were measured using a glucose detection kit (Abcam, 102517) following the manufacturer's instructions.

### Protein tagging and constructs

Protein tagging was performed as described by Bähler et al. (1998), using the primers listed in Table S3, except that plasmids were equipped with a ‘happy linker’ sequence between gene and fluorophore where indicated, as for EB1 by Jankovics and Brunner (2006). Monomeric mGFP(A206K) was generated from the routinely used GFP(S65T) by site-directed-mutagenesis of alanine 206 to a lysine at the dimer interface using forward (5′-AGGTCGACGGATCCTTGGAG-3′) and reverse (5′-GATCTTTCGAAAGTTTAGATTGTGTGGACAGGTAATGGTTGTCTGG-3′) primers, and insertion into the original plasmid using restriction enzymes BamHI and BstBI (Snapp, 2003).

### Microscopy

Live imaging was performed at room temperature on a spinning disc microscope (Zeiss Axio Observer Z1, Yokogawa CSU-X), using 63× and 100× NA 1.4 oil plan apo objectives, Andor iQ2.9 software, Andor Neo sCMOS and iXon3 EMCCD cameras (Andor Technology, Belfast, UK), 488 nm and 561 nm laser excitation, and 525/50 BP and 568 LP emission filter sets. For standard fluorescence, z-stacks at 0.5 µm steps were acquired unless stated otherwise.

DIC microscopy of starved cells was performed on glass bottom dishes (Bioswisstec, Schaffhausen, Switzerland; 5160) coated with poly-L-lysine (2 mg/ml, Merck, P1399). In the dishes, we constructed a chamber by adding a cover slip on top of three parafilm strips acting as spacers. The dishes were then placed for 2 s on a heating plate at 100°C, which partially melted the strips, such that they glued the coverslip to the dish. The, thereby, formed chamber was filled with cultured cells (∼30 µl) by pipetting into the gap between the parafilm strips. To extract LD trajectories from DIC movies, cells were centrifuged at low speed (300 rpm, Multifuge 1S-R, Thermo Fisher Scientific) and imaged immediately (5–15 min after mounting), as prolonged residence in the chamber causes artefacts. For each movie, 100 image frames were acquired at four frames per second.

LD trajectories of cells exiting starvation (Fig. 1E) were extracted from DIC movies using lectin-coated glass bottom dishes (*Griffonia simplicifolia* lectin1; Vector laboratories, Burlingame, California; L-1100). A first movie was taken from cells in Edinburgh minimal medium without glucose (EMM0G) immediately before glucose addition. After addition of 2% glucose, movies were taken for up to 60 min.

Live cell imaging for PCC quantification was done on lectin-coated glass bottomed eight-well (ibidi, Martinsried, Germany; 80827) or ten-well slides (Greiner Bio-One, Kremsmünster, Austria; 543079) after centrifugation at 174 ***g***. 1 mg/ml BODIPY (BODIPY 493/503; Thermo Fisher Scientific; D3922) was dissolved in DMSO and used at a final concentration of 4 µg/ml in EMM2 or EMM0G for exponentially growing or starved cells, respectively. The dye phloxine B (Merck, P4030) was dissolved in water to 5 mg/ml, diluted to 100 µg/ml in water, and used at a final concentration of 10 µg/ml. When imaging, we first acquired a single focal-plane image of red fluorescent phloxine B followed by three single focal-plane images of green fluorescent BODIPY with a time interval of 42 s.

The movies for cox4p-GFP particle tracking were made of 300 frames taken at four frames per second in chambers as for DIC imaging.

Protoplasts in high sorbitol were imaged using DIC microscopy on lectin-coated glass-bottomed ten-well slides after centrifugation at 174 ***g***. Protoplasts kept in low sorbitol concentration were transferred to a lectin-coated imaging chamber (see above), sealed off with VALAP (Vaseline, lanolin, paraffin; 1:1:1) to prevent dehydration, and imaged immediately.

### Digestion of the cell wall

Protoplasts in high sorbitol were generated by enzymatic digestion of cell wall, with slight alterations to a previously published protocol (Kelly and Nurse, 2011). To form protoplasts, cells were incubated at 25°C with 5 mg/ml Zymolyase 20-T (MP Biomedicals) plus 5 mg/ml lysing enzymes from *Trichoderma Harzianum* (Merck; L1412) in 500 μl E-buffer +1.2 M sorbitol in a 2 ml Eppendorf tube for 1 h on a rotor at 25°C unless stated otherwise.

Protoplasts in low sorbitol were generated by washing cells in E-buffer+0.5 M sorbitol, centrifuged at minimal speed for 5 min, and resuspended in 50 μl E-buffer+0.5 M sorbitol plus cell wall-digesting enzymes.

For DED, protoplasts were generated in continuous presence of 20 mM 2-deoxy glucose and 10 mM antimycin A in E-buffer as described in (Munder et al., 2016).

### Acquisition and analysis of FLIP

FLIP experiments were performed on cells mounted to an imaging chamber sealed with VALAP (see above). Imaging was done at room temperature on a spinning disc microscope (Nikon Eclipse Ti, VisiScope system, Yokogawa W1) using a 60× water objective, VisiView software, and an Andor EMCCD camera (iXon Ultra 888 back illuminated). A z-stack of three planes (1 µm step size) was acquired every second for 100 s while a small region with 1.12×1.12 µm size near one cell pole was bleached every 5 s. The mean fluorescence intensity loss of a reference region at the opposite pole was then extracted using Fiji. The analysis was done using Matlab, as described in (Bancaud et al., 2010). The signal was normalised to the last pre-bleach time point. For each condition, 30 cells were analysed – ten cells each in three independent experiments.

### Electron microscopy

Cells were high-pressure frozen in solution (reviewed by McDonald et al., 2010) using a Wohlwend Compact-2 high-pressure freezer (Martin Wohlwend AG, Sennwald Switzerland). *S. pombe* samples destined for plastic section microtomy were freeze-substituted in 0.1% glutaraldehyde and 1% uranyl acetate in acetone for 48 h and warmed from −90°C to −50°C in 8 h at 5°C per hour. Cells were then washed by acetone for three times and infiltrated in HM20 solution (25%, 33%, 50%, 67%, 75%, 100% in acetone) (Lowicryl HM20 Embedding Kit, Electron Microscopy Science, Hatfield, PA) over 5 days using Leica EMAFS (Leica, Vienna, Austria). Samples were then polymerised to blocks under a Leica EMAFS UV light unit for 72 h.

Plastic blocks were cut into ribbons of 80 (for single projection images) 250-nm-thick sections (for tomographic reconstructions), depending on the questions asked, by using a Leica Ultracut microtome (Leica Inc., Vienna, Austria) or a Diatome Ultra 45° diamond knife (Diatome AG, Biel, Switzerland). Ribbons were collected on formvar-coated Cu-Rn grids (Electron Microscopy Science, Hatfield, PA) or carbon film finder grids (Electron Microscopy Science), immuno-labelled (optional), stained by uranyl acetate (2% uranyl acetate in 70% methanol) for ∼4 min and Reynold's lead citrate for ∼2 min ; the staining time was adjusted to the thickness of the sections.

Individual pictures of plastic sections, mostly used as a control, were acquired with a FEI Philips CM100 TEM and AMT 2K× bottom-mount digital camera.

### Treatment with latrunculin B

Latrunculin B (LatB; the toxin of *Latrunculia magnifica*; Merck; 428020) was added from a stock solution of 10 mM in DMSO to a final concentration of 100 µM (1% DMSO). Control cultures were treated with 1% DMSO. For the short-term effect of this LatB concentration, the stock solution was diluted in EMM2 for exponentially growing cells and EMM0G for SD3 and SD6 cells. For incubation with LatB for 3 days, starved cultures were split in half at SD3. One half was supplemented with LatB, the other with DMSO to serve as control. Both cultures were incubated at 25°C on the shaker for another 3 days.

### Western blotting

After culturing, 0.5×10^8^ cells (two OD_595_ equivalents) were harvested by centrifugation and resuspended in 200 μl lysis buffer (1% SDS, 8 M urea, 10 mM MOPS pH 6.8, 10 mM EDTA, 0.01% Bromophenol Blue) containing 100 μl glass beads and a protease inhibitor cocktail (Promega) (Marguerat et al., 2012). The cells were disrupted by vortexing for 3×40 s in a Fastprep FP12 cell disrupter (Thermo Fisher Scientific). The protein samples were denatured and reduced with 5 mM DTT for 10 min at 65°C and clarified by centrifugation before SDS-PAGE and western blot analysis using 1:1000 anti-GFP mouse monoclonal antibody (11814460001, Roche) or anti-actin antibody mouse monoclonal antibody (224-236-1, DSHB).

### Image analysis

Routine image processing was done using Fiji/ImageJ. Deconvolution was done using Huygens software (Scientific Volume Imaging) on image stacks acquired using Nyquist criteria. Plots were made using Matlab (MathWorks). Optimal sample sizes were not explicitly calculated.

### LD trajectories from DIC movies

The DIC movies (100 frames, four frames per second) were stack registered (Fiji plugin ‘StackReg’). LDs were tracked with the Fiji plugin ‘Manual Tracking with TrackMate’ (settings for semi-automated tracking: Quality threshold: 0.2, Distance tolerance: 0.1, Max nFrames: 0). The LDs were manually seeded in the first time frame, and the trajectory was considered when the particle could be tracked for >95/100 frames. The manually seeded first trajectory point was excluded from the final trajectory, such that all LD positions were automatically detected. Trajectories were plotted using Matlab.

### Mean square displacement of mitochondria

The cox4p-GFP-labelled mitochondria were tracked using the Mosaic particle tracking plugin in Fiji (Mosaic Toolsuite, (Sbalzarini and Koumoutsakos, 2005); Settings: radius: 4, cut-off: 0, per/abs: 2, link: 3, displacement: 2). Subsequent analysis was done in Matlab (Mathworks). Only trajectories of >160 frames were considered, and the mean square displacement up to a time lag of 40 frames was computed. The time-averaged mean square displacement for each particle was plotted as a colour-coded histogram and, additionally, the ensemble-averaged mean is shown.

### PCC quantification of LD motion

Cell segmentation: the phloxine B signal was log-transformed and background-subtracted in Fiji (Mosaic ToolSuite). Using pixel classification in ilastik the inside of the cell was separated from the outline and the background. Phloxine B-filled; therefore, dead cells were marked as a separate group and excluded from subsequent analysis. Cell insides were segmented in Cellprofiler and used as seeds to segment living cells (Fig. S1D). Subsequently, the three BODIPY images, taken at 42-s intervals (t1 to t3), were stack-registered in Fiji (StackReg). We included additional procedures to account for high variability of BODIPY signal intensity amongst individual cells in deep starvation as well as significant differences of entire populations between different starvation days, with particularly low signals at SD2 and SD3 (Fig. S1F). In addition, for some unknown reason, BODIPY signal intensity gradually increased during imaging, with a main increase between t1 and t2 (Fig. S1F). As a result, the software generally detected LDs most efficiently at t2 and t3, which is why we used these timepoints to determine the PCC values. Because absolute signal intensities are irrelevant when extracting LD dynamics, we equalised fluorescence on our images by performing a log transform and background subtraction (Mosaic ToolSuite). Pixel classification in Ilastik further improved equalisation. This generated pseudo images, in which pixel values represent the probability to belong to an LD instead of the actual fluorescence intensities (Fig. S1G). From these images, we computed the PCC for all pixels of individual cells between t2 and t3 using Cellprofiler. We presented individual PCC values as dot plots using the function plotSpread from the MathWorks File Exchange (https://ch.mathworks.com/matlabcentral/fileexchange/37105-plot-spread-points--beeswarm-plot-). We overlaid a boxplot indicating the median and the 25th to 75th percentile of the values. When several experiments were pooled, the mean of the medians of the single experiments was plotted in blue. As a measure for the variance between individual experiments, blue error bars indicate the 95% confidence interval of the medians. This variance does not describe the variance of the individual medians and might thus underestimate the true variance of the median.

### Cell size measurements

To compare the size of cells cultured in standard EMMLG and further incubated in hypertonic buffer containing 1.2 M sorbitol (Figs 2E,F and 6D; Fig. S4), cell length and width were measured manually from DIC images by using Fiji. Subsequently, we extracted the means of cell length and cell width measured from three independent experiments each, for cells cultured in standard EMMLG and those incubated in hypertonic buffer containing 1.2 M sorbitol. To estimate the variance (*dL*^2^, *dW*^2^) of the three experiments, bootstrapping was performed (999× resampling of each individual experiment), leading to 999 new means from bootstrapped samples. The mean cell volume, *V*, was calculated as a cylinder plus a ball from the mean of the bootstrapped means of measured length and width (*L*, *W*) as follows(1)
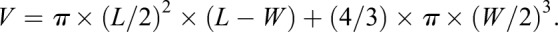
The variance of the cell volume (*dV*^2^) was estimated by Gaussian error propagation with(2)
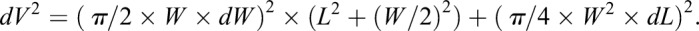
To define the amount of cell shrinkage after transfer to the hypertonic buffer, we divided the mean of the bootstrapped means of cells in hypertonic buffer (*S*) by the mean of cells cultured in EMMLG (*E*) for length, width and approximated volume. The variance (*dR*^2^) of the ratio *R*=*S*/*E* was calculated by Gaussian error propagation using(3)
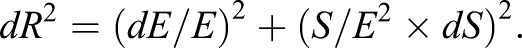
The mean and 95% confidence interval of these normally distributed fractions are shown in Figs 2F and 6D.

Measurements of cell length and width using phloxine B signal was done in an automated fashion as described above.

## Supplementary Material

Supplementary information
