# Supplementary material for: Reversible solidification of fission yeast cytoplasm after prolonged nutrient starvation
Source: J Cell Sci. 2019 Nov 8;132(21):jcs231688. doi: 10.1242/jcs.231688 (PMC6857596; doi:10.1242/jcs.231688)
Supplement: Supplementary information [file joces-132-231688-s1.pdf]

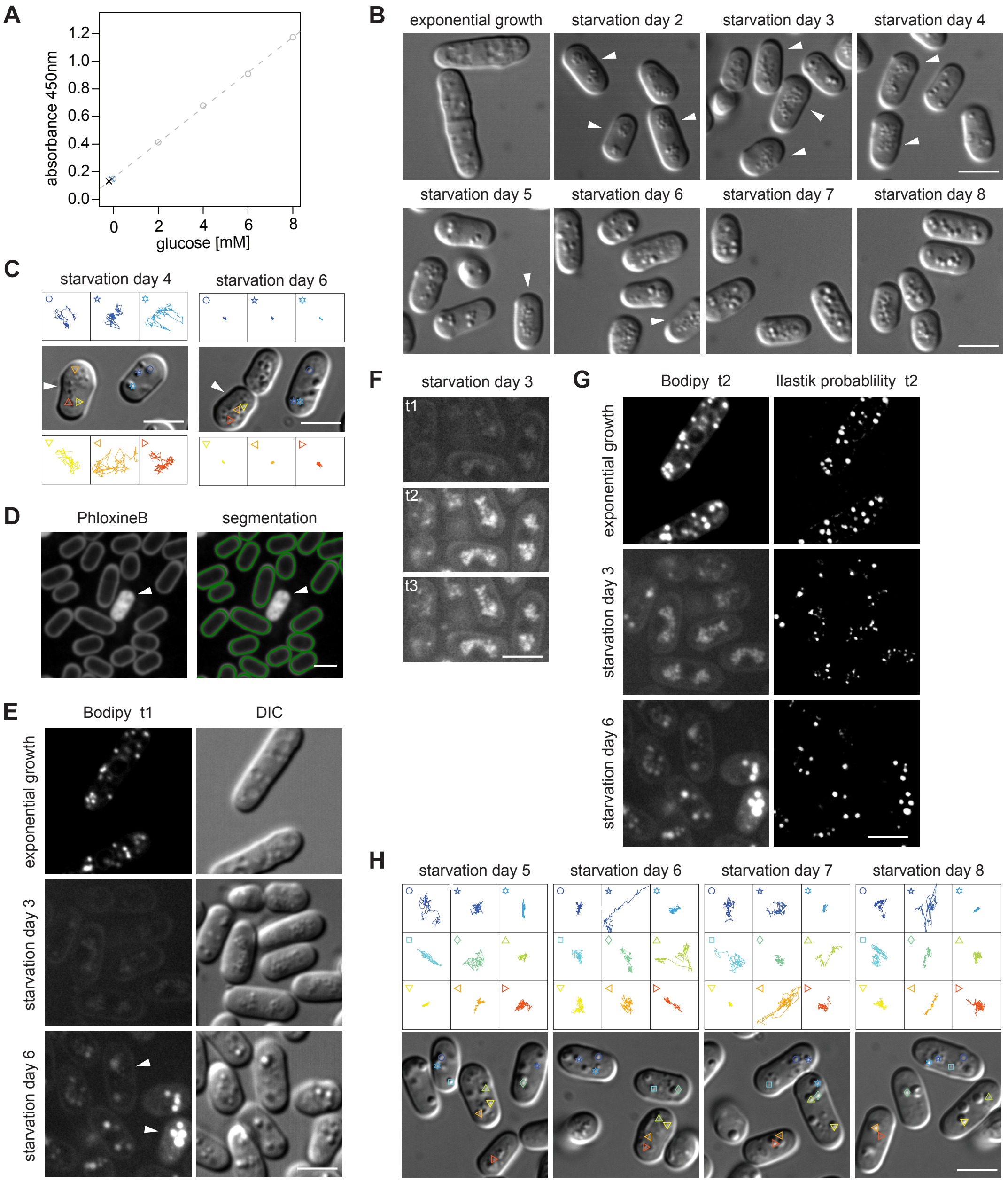

Journal of Cell Science • Supplementary information

Figure S1 - LD morphology and mobility analysis in starvation.

A Glucose measurement, comparing filtered medium of an SD2 cell culture (black cross) with culturing medium without added glucose (blue cross) and a standard curve of glucose in water at 0, 2, 4, 6, 8 mM (grey circles) with the calculated curve fit (R^2=0.9995).

B LD composition changes from evenly distributed droplets in exponentially growing cells to grape-like LDs (white arrowheads) in cells on SD2. From SD3 to SD5 cells increasingly contain fewer but bigger LDs.

C LD trajectories as in Fig 1A, comparing cells containing grape-like LDs (white arrows; orange trajectories) and cells containing bigger LDs (blue trajectories). The left panel shows cells on SD4, the right panel on SD6.

D Images show Phloxine B-labelled cells (upper panel) as used to segment cells. Segmented cell outlines are shown in green (lower panel). Dead cells internalized Phloxine B (white arrow) and were excluded from segmentation and subsequent analysis.

E The upper panels show the Bodipy signal in the cells of the DIC images in the lower panels, during exponential growth, on SD3 and on SD6. White arrows depict individual cells with an extreme difference in Bodipy signal intensity.

F Bodipy-labelled cells on SD3 imaged at 3 consecutive time points (t1-t3), separated by 42s.

G Same cells as in (E) showing the Bodipy image pixels in the upper panels and in the lower panels the respective pseudoimages resulting from classification with Ilastik. The pseudoimages show the probability of pixels to belong to LDs as was then used to compute the CC between two imaged time points. Note that contrast settings for the same pictures differ in (D) and (F).

H LD trajectories as in Fig 1A, of a cell population in which no motion arrest occurred up to SD8.

Scale bars: 5 µm.

Journal of Cell Science • Supplementary information

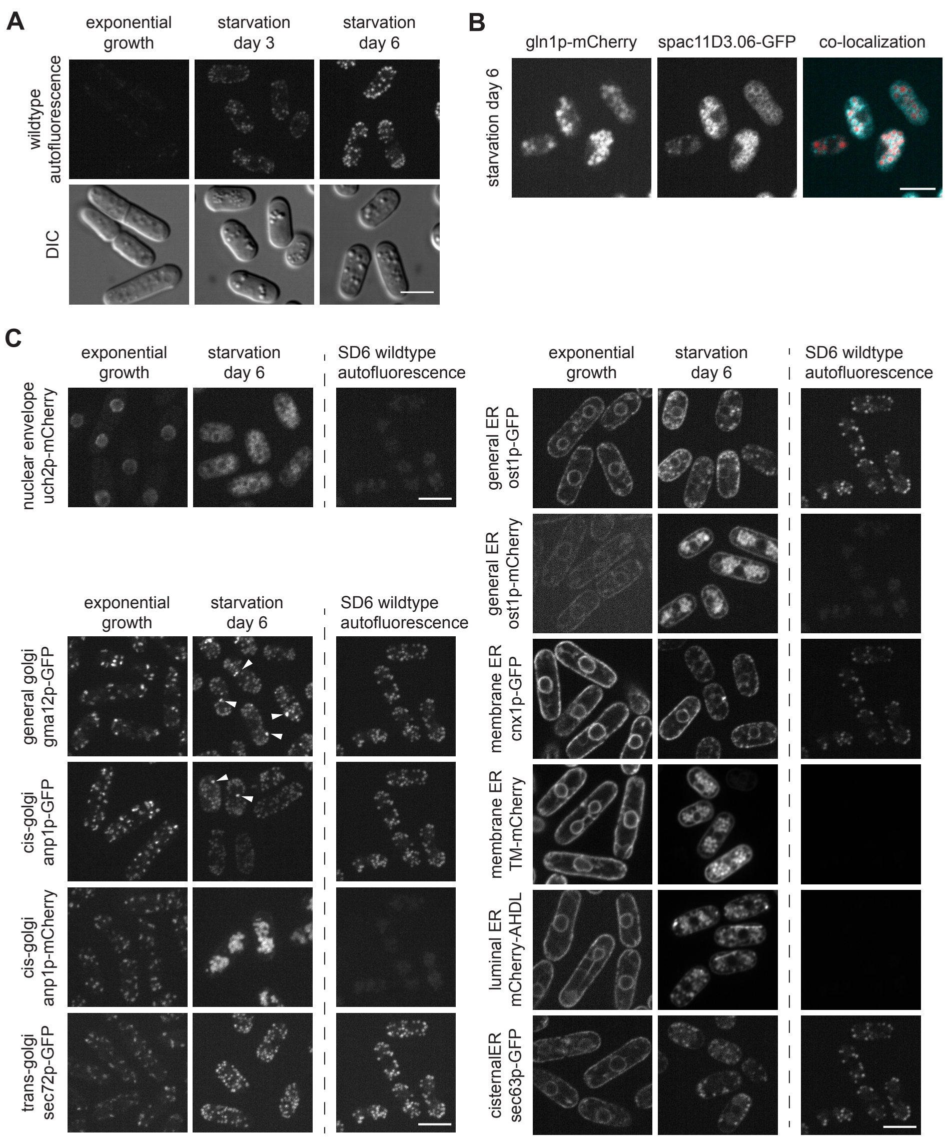

Figure S2 - Autofluorescence and organelle markers in deep starvation.

A Autofluorescence (excitation 488nm, emission 525/50) of wild type cells in exponential growth, on SD3 and SD6. Images show maximum intensity projections.

B Single focal plane images showing double labelling of gln1p-mCherry and the vacuolar marker Spac11D3.06-GFP.

C Images show fluorescence of markers for the indicated subcellular structures in exponentially growing cells and on SD6. The unspecific signal portion can be estimated from comparison to the autofluorescence from a SD6 wild type cell without fluorescent tag with the same imaging and contrast settings (to the right of dashed line). All images are maximum intensity projections.

Scale bars: 5 µm.

Journal of Cell Science • Supplementary information

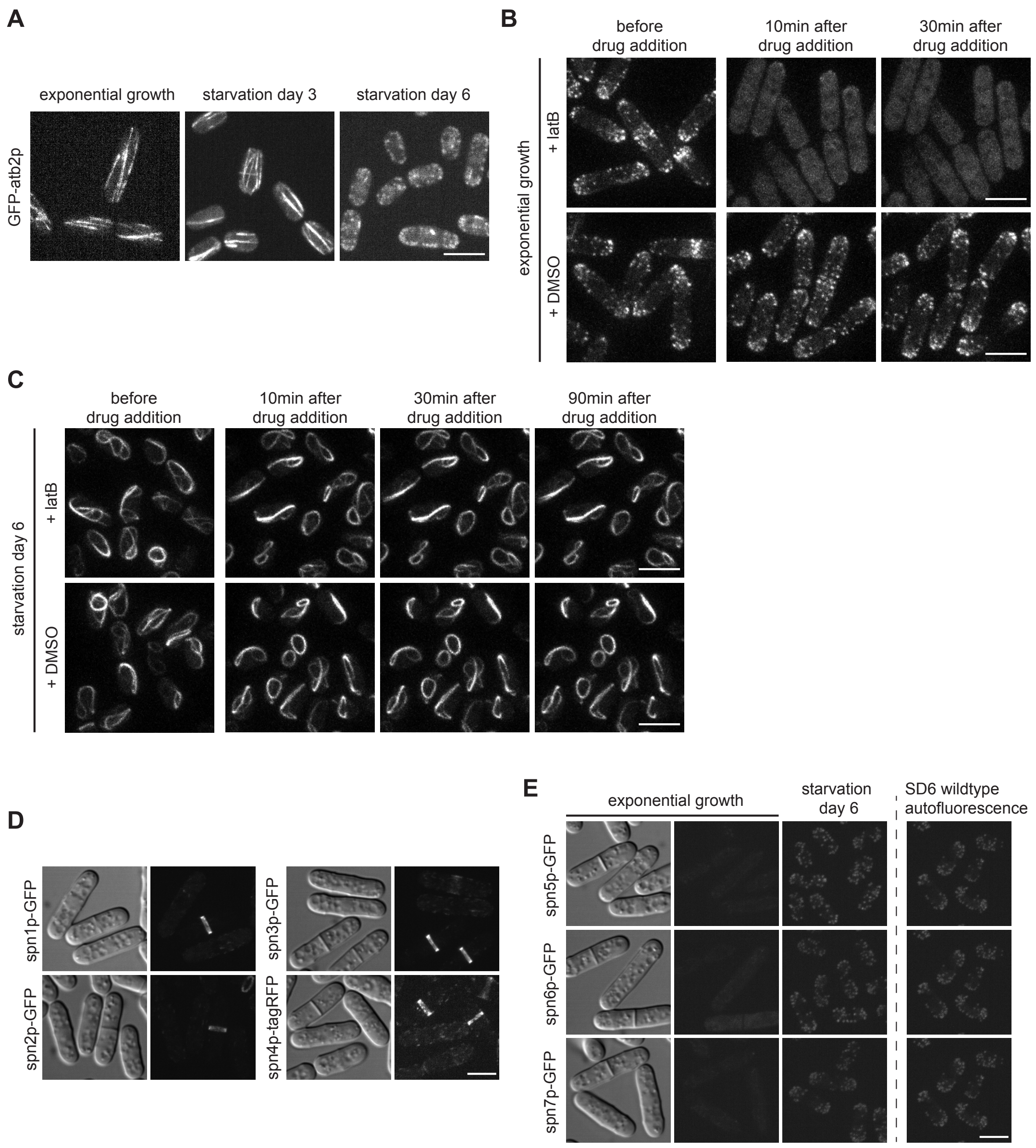

Figure S3 - Cytoskeleton in deep starvation.

A Microtubules visualized by GFP-atb2p during starvation.

B Effect of 100 µM LatB/DMSO on F-actin in exponential cells. Images represent maximum intensity projections.

C Same as (B) for SD6 cells.

D Spn1-4p localization in exponentially growing cells.

E GFP-tagged spn5-7p in exponentially growing cells (left; corresponding DIC images show cell location) and on SD6 (middle). The unspecific signal portion can be estimated from comparison to the autofluorescence from a SD6 wild type cell without fluorescent tag with the same imaging and contrast settings (to the right of dashed line). Fluorescence images represent maximum intensity projections.

Scale bars: 5 µm.

Journal of Cell Science • Supplementary information

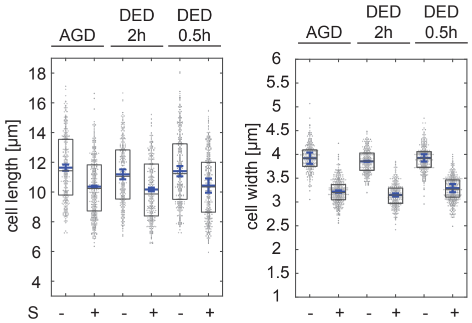

Figure S4 - Hypertonic environment induces volume loss in acutely energy depleted cells. Cell length and width of AGD and DED (2 and 0.5 h) cells in standard culturing medium (-S) or in 1.2 M sorbitol containing buffer (+S) from 3 independent cell populations each, measured manually from DIC images (n = 341, 452, 324, 340, 371, 442). The blue line represents the mean of 3 independent experimental means. Error bars show the 95% confidence interval.

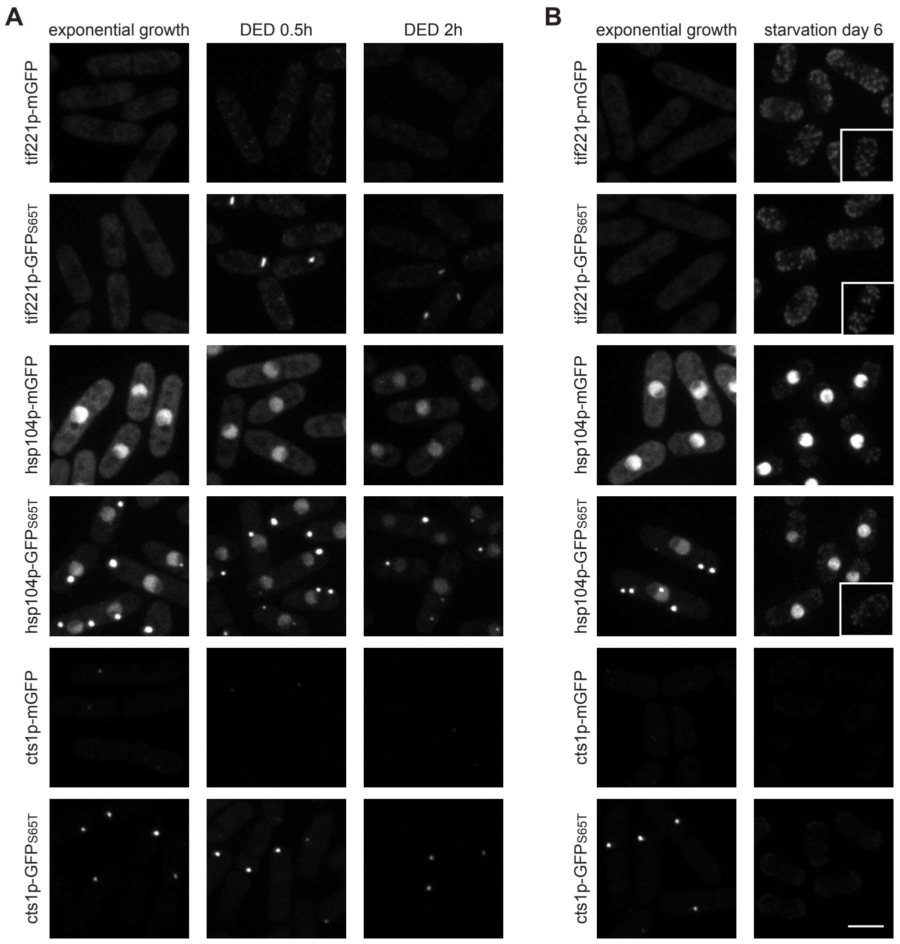

Figure S5 - GFP(S65T)-dependent protein assemblies.

A Images show fluorescence signal of the indicated fusion proteins with mGFP and GFP(S65T) respectively in exponentially growing cells (left panels) and DED cells (right panels) incubated for 0.5 or 2 h prior to imaging. Note that corresponding fusion proteins with mGFP and GFP(S65T) show the same imaging and contrast settings.

B Images show fluorescence signal of the indicated fusion proteins in exponentially growing cells (left panels) and SD6 cells (right panels). The unspecific signal portion can be estimated from comparison to the autofluorescence from a SD6 wild type cell without fluorescent tag with the same imaging and contrast settings (insets). Images are maximum intensity projections in all panels. All observations were confirmed in 2 independent experiments.

Journal of Cell Science • Supplementary information

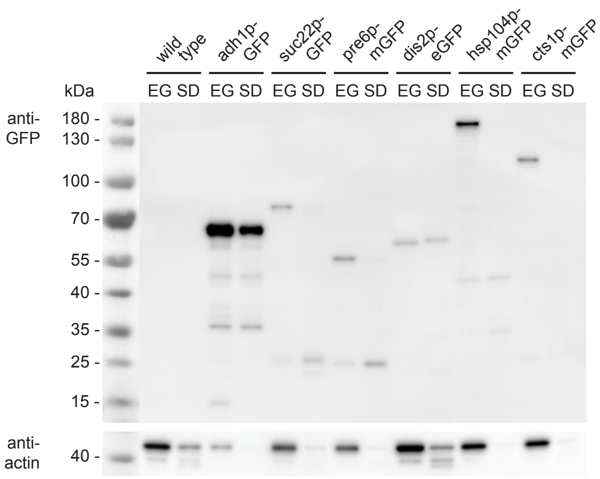

Figure S6 - Protein levels in deep starvation

Western blot of wild type control cells and cells expressing the indicated fusion proteins. anti-GFP (upper panel) and anti-actin antibodies (lower panel) were used for cells in exponential growth (EG) and cells at SD6. For better visualization loading of the different strains was adapted from left to right as follows: 10x, 1x, 5x, 2.5x, 10x, 2x, 2x.

Scale bars: 5 µm.

Journal of Cell Science • Supplementary information

Table S1: Strains used in this study

Strains	Genotype	Source
DB404	h- sec63-GFP::kanMX6 ura4-D18 leu1-32	(Vjestica et al., 2008)
DB558	h- wild-type	(Leupold, 1950)
DB559	h+ wild-type	(Leupold, 1950)
DB933	h- nup85-GFP::kanMX6 ade6-M216 leu1-32	This study
DB2003	h+ cnx1p-HL-GFP::kanMX6 ura4-D18 leu1-32 ade6-M216 **	This study
DB2057	h- SV40-GFP-atb2::leu2 leu1-32	(Pardo and Nurse, 2005)
DB2400	h+ anp1-GFP::ura4 ade6-216 ura4-D18 leu1-32	(Vjestica et al., 2008)
DB2401	h+ sec72-GFP::ura4 ade6-216 ura4-D18 leu1-32	(Vjestica et al., 2008)
DB2402	h+ anp1-mCherry::ura4 ade6-216 leu1-32 ura4-D18	(Vjestica et al., 2008)
DB2403	h+ uch2-mCherry::ura4 ade6-216 leu1-32 ura4-D18	(Vjestica et al., 2008)
DB2404	h- ost1::GFP-ura4 ura4-D18 leu1-32	(Vjestica et al., 2008)
DB2405	h+ ost1-mCherry::ura4 ade6-210 leu1-32 ura4-D18	(Vjestica et al., 2008)
DB3287	h- spn1::kanMX6	(Wu et al., 2010)
DB3293	h- spn2::ura4+ ura4D18	(An et al., 2004)
DB3297	h+ spn2-GFP::kanMX6 *	(An et al., 2004)
DB3310	h+ spn3-GFP::kanMX6 *	(An et al., 2004)
DB3324	h- spn4::kanMX6 *	(Wu et al., 2010)
DB3326	h+ spn5-HL-GFP::kanMX6	This study
DB3340	h+ spn6-HL-GFP::kanMX6	This study
DB3410	h- leu1-32::pAct1-Lifeact-GFP::leu1 *	(Huang et al., 2012)
DB3422	h- spn1-GFP::kanMX6 *	(Wu et al., 2010)
DB3426	h+ spn3::ura4 ura4-D18	(An et al., 2004)
DB3455	h+ spn7-GFP::kanMX6 *	(Onishi et al., 2010)
DB3587	h- spn4-tagRFP::kanMX6	This study
DB3623	h- Pbip1-GFP-AHDL::leu1 ura4-D18 leu1-32 ade6	(Zhang et al., 2010)
DB3624	h- Pbip1-mCherry-AHDL::leu1	(Zhang et al., 2010)
DB3726	h- cox4-GFP::leu2 leu1-32 *	(Fu et al., 2011)
DB3856	h- hsp104-mCherry::kanMX6 (hsp104***)	(Coelho et al., 2013)
DB4192	h- gma12-GFP::ura4 ura4-D18 *	(Wang et al., 2002)
DB4233	h- cps8-188	(Ishiguro and Yamada, 1993)
DB4672	h+ Pnmt1-TM-mCherry::leu	(Zhang et al., 2012)
DB5013	h- atg8::kanMX6 *	Bioneer M-4030H-U5 (Kim et al., 2010)
DB5018	h- atg1::kanMX6 *	Bioneer M-4030H-U5 (Kim et al., 2010)
DB5160	h- cts1-HL-GFP::kanMX6 (ura7***)	This study
DB5162	h- pre6-HL-mGFP::kanMX6 (pre6***)	This study
DB5209	h+ suc22-GFP (rnr4***)	(Vejrup-Hansen et al., 2014)
DB5310	h- gln1-mCherry::natR (gln1***)	(Coelho et al., 2014)
DB5315	h+ dis2-NEGFP::ura4 ura4-D18 * (glc7***)	(Alvarez-Tabarés et al., 2007)
DB5320	h+ adh1-GFP::kanMX6 * (adh2***)	(Sigova et al., 2004)
DB5380	h+ cts1-mCherry::kanMX6 *(ura7***)	(Coelho et al., 2014)
DB5381	h- hsp104-GFP::kanMX6 * (hsp104***)	(Coelho et al., 2013)
DB5470	h- tif221-HL-GFP::kanMX6 (gcn3***)	This study
DB5730	h+ nmt1:GFP-HL-Spac11D3.06::kanMX gln1-mCherry::natR	This study
DB5869	h- tif221-HL-mGFP::kanMX6 (gcn3***)	This study
DB5870	h- tif221-HL-mCherry::kanMX6 (gcn3***)	This study
DB5871	h- cts1-HL-mGFP::kanMX6 (ura7***)	This study
DB5872	h- hsp104-HL-mGFP::kanMX6 (hsp104***)	This study

* auxotrophic alleles were eliminated by crossing to wild type (DB559); ** HL = “happy linker” (Methods);
*** *S. cerevisiae* orthologs

Journal of Cell Science • Supplementary information

Table S2: Proteins tested for assembly formation

gene name	product https://www.pombase.org/	GO cellular component https://www.pombase.org/	Fusion protein localisation								
			mCherry			mGFP			GFP(S65T)		
			EG	DED 0.5/2h	SD6	EG	DED 0.5/2h	SD6	EG	DED 0.5h/2h	SD6
adh1	alcohol de–hydrogenase	-	na	na	na	na	na	na	C, N	C, N	(C, N)
suc22	ribonucleotide reductase small subunit	cytosol, nucleus, ribo-nucleoside-diphosphate reductase complex	na	na	na	na	na	na	N	N	N
pre6	20S proteaso-me complex subunit alpha 4	cytosol, nuclear periphery, proteasome core complex	na	na	na	C, N	C, N	(C, N)	na	na	na
dis2	serine/threoni-ne protein phosphatase PP1	cytosol, nucleus, cell tip, nuclear chromatin, cell division site, DPS complex	na	na	na	na	na	na	C, N	C, N	C, N
gln1	glutamate-ammonia ligase	cytosol, nucleus	C, N	C, N	nd	na	na	na	na	na	na
tif221	translation initiation factor eIF2B alpha subunit	cytosol, eukaryotic translation initiation factor 2B complex	C	C	(C)	C	C	(C)	C	A	(C)
hsp104	heat shock protein	cytosol, nucleus, nuclear envelope	C, N	C, N	N	C, N	C, N	N	A	A	N
cts1	CTP synthase	cytosol	C, N	C, N	nd	C, N	C, N	nd	A	A	nd

C = cytosolic, N = nuclear, A = assemblies present, na = not analysed, nd = not detectable

Journal of Cell Science • Supplementary information

Table S3: Primers used in this study

DB3340	DPE872	CTGAATATCACGAAAGGATCCGTGCTTTGGAGACCCAAATTGAAAGTTTGAAAAGTTACAAGGGCCGCGGTCATAAAAAAatccttggagctccttcagga
	DPE873	AATTGCAAATTTAGTAAGAAAAAGCCATCAGATGAGCAAATAAAAGGAGATGGAAAAGTTAAAAGTTTGACTTGAGACTTgaattcgagctcgtttaaac
DB933	DPE 275	GTCAACTGTAAAGGACCAGCAGCTTTTACTATCCATTCATGAGCGTCTTTCTTCTGCGATATCATGGTATTTTCTTCACTTAAAAAAAatccttggagctccttcagga
	DPE 274	GTATCTTAATAAAAACATGTATGAAGCTTCTATGTTACAGAAAGATTAAAATGTCAAGTAACAGAAATAGCCTAATTTAAATCCCgaattcgagctcgtttaaac
DB2003	DPE739	TAAGCAAGAGACTGAGACTGAGAAGATAGACGTTTCTTACGCTCCCGAAACTGAATCACCAACTGCGAAGAATGAAGACAatccttggagctccttcagga
	DPE740	GATAGTACTACCCACGATTTATAAATTCATAGTCTATTTATTGATATTACTCATAATAAGAACTAGAGAAAACAgaattcgagctcgtttaaac
DB5160 and DB5871	DPE1495	ATTTTAACGCTGAATCTGCCTTAGCTGACATGAATGACTCTGTTGAAGTTACTGAAGAAGCCACTGTCGTCACCATCAGT atccttggagctccttcagg
	DPE1496	CACCCCAGAACCCAATTTTTCCTATAGATAAAGAAAAACACACCAACAAACACACATTATTTCCTAATTCCCGGAATCCCgaattcgagctcgtttaaac
DB5162	DPE1450	AAGATGAGAAAGAAGCTGAAGCTGCTCGTAAAAAGAGTGGCCGTTCTGCCCCTGGAGTCTCTACAACTTCTACGATTCAAatccttggagctccttcagg
	DPE1451	CAAAAGGGAAAAGACATATGAACCTTATAAACAAGAAATTCTTAAGTCGGTTTGCATGTAATGAAATAAAAGAGGTATCAgaattcgagctcgtttaaac
DB5470	DPE1535	TTGTCTCAGGTCTCATTACCGATTTAGGGATCATTGATTCGAAGAGTGGGGTAAGCGAAGAGCTAATTAAATTGTATCTTatccttggagctccttcagg
	DPE1536	AAGACTTATGAGAAATTTAAGTCAACTCAAAGTACAATCTATTCATATTTTATTTTTAAGATCAGGAGAATCTGATTG gaattcgagctcgtttaaactg
DB5730	DPE1473	GGTTCCATCCGTTCAATCAATATGATAAAAGCTTAGTAAACTTTTATTAAAGGAAAATTTGAACCTTCGGTGAACAGACAgaattcgagctcgtttaaac
	DPE1474	GAGTTTTGCAAGGCATATCCAAGGATTACCGGAGCTGAATTTATCAAAAGGTATTTCACCTCTGTAAGTGGTCTACCCATaattaaccccgcaggtccac
DB5869 and DB5870	DPE1535	TTGTCTCAGGTCTCATTACCGATTTAGGGATCATTGATTCGAAGAGTGGGGTAAGCGAAGAGCTAATTAAATTGTATCTTatccttggagctccttcagg
	DPE1536	AAGACTTATGAGAAATTTAAGTCAACTCAAAGTACAATCTATTCATATTTTATTTTTAAGATCAGGAGAATCTGATTGgaattcgagctcgtttaaactg
DB5872	DPE1755	ATCATGAAGCTAATGCAAACGGCTCTGCTGATATTGACATGGATGGTATTGACGACGATGTTAATGATGAAGAATTGGAAatccttggagctccttcagg
	DPE1756	TGTACTTAGCTTTTGTTAAATTGATTCTATCTATTCGATAAAGTTCATTGAACATTTTAATGAGAAAACAGTACTTCCgaattcgagctcgtttaaactg

Journal of Cell Science • Supplementary information

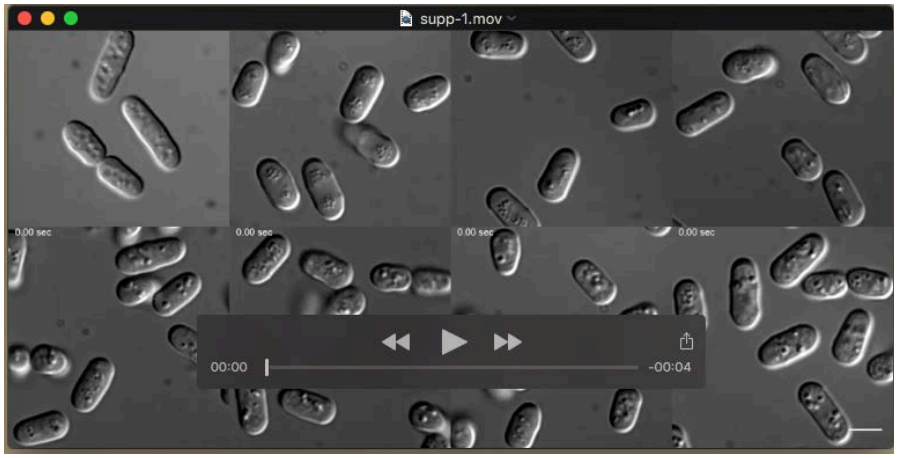

Movie 1 - Motion arrest of LDs in deep starvation

25sec DIC movies (4frames/sec) of cells in exponential growth and starvation days 2-8. Upper panels from left to right show cells in exponential growth and on starvation days 2, 3 and 4. Lowers panels show cells at starvation days 5, 6, 7 and 8. Scale bar: 5µm.

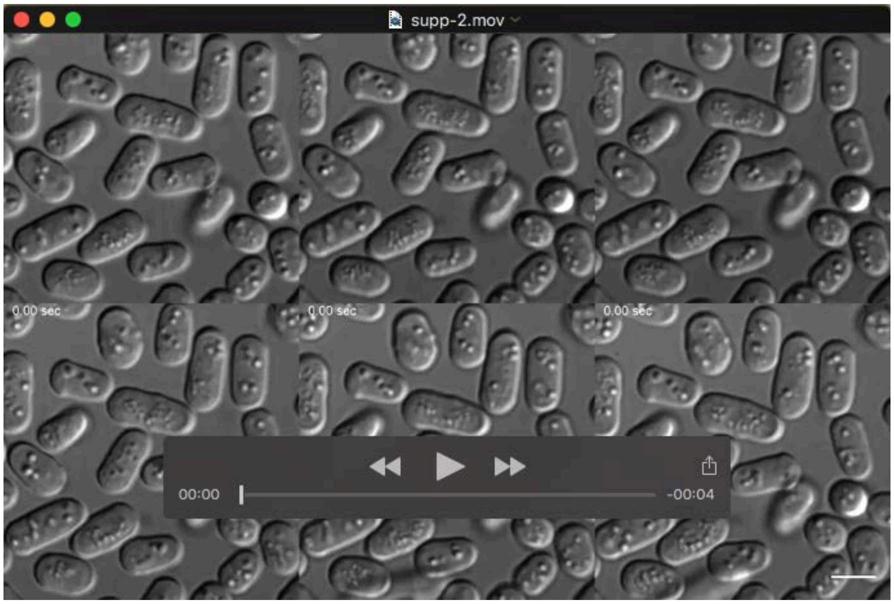

Movie 2 - CF exit following glucose addition

25sec DIC movies (4frames/sec) of cells on starvation day 5 before and after glucose addition. Upper panels from left to right show cells before glucose addition and 5min or 10min after glucose addition. Lower panels show cells 15min, 30min and 60min after glucose addition. Scale bar: 5µm.

Journal of Cell Science • Supplementary information

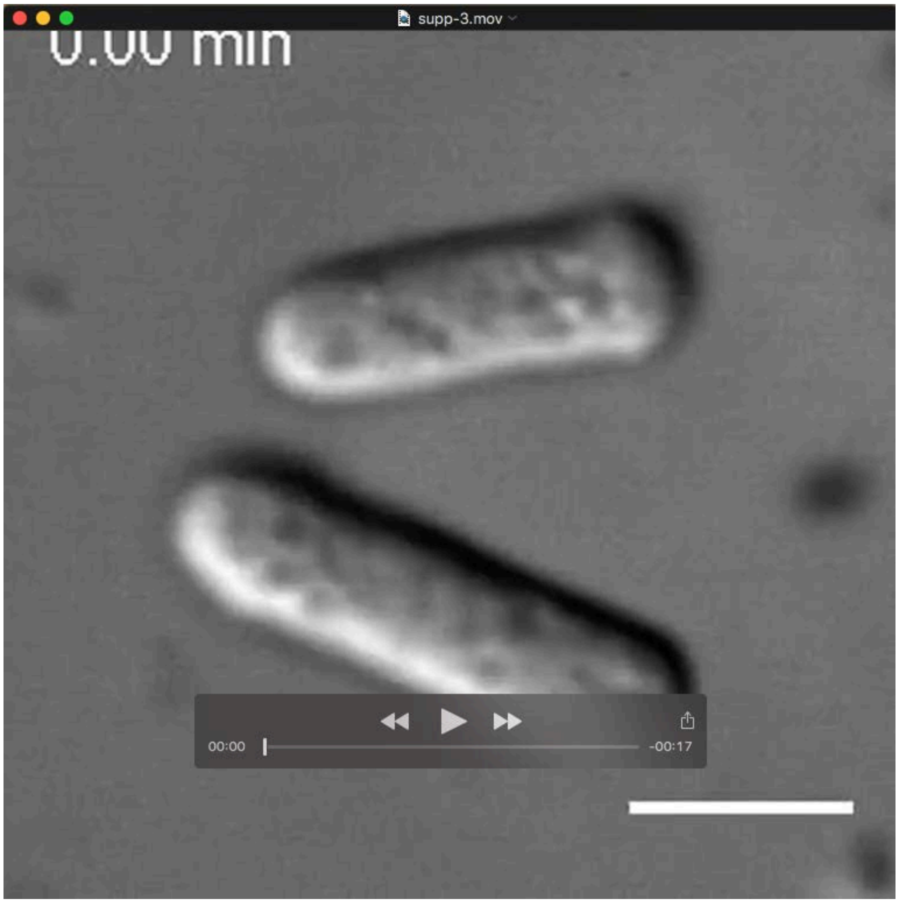

Movie 3 - Protoplasting of exponentially growing cells in hypertonic conditions

DIC movies (1frame/10sec) of protoplast evasion from the cell wall of exponentially growing cells in a dish. Cells are being incubated in 1.2M sorbitol containing cell wall digesting mix. Note that the bottom cell is undergoing cytokinesis. Scale bar: 5µm.

Journal of Cell Science • Supplementary information

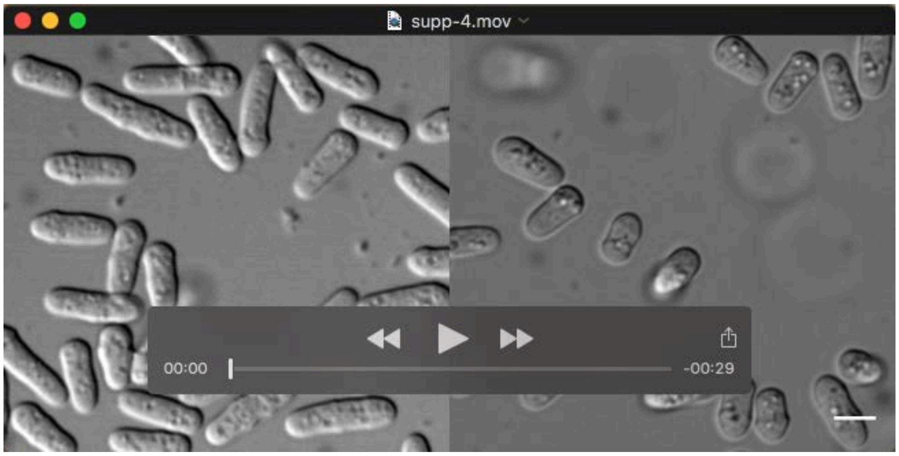

Movie 4 - Protoplasting of cells in hypotonic conditions

DIC movies (1frame/10sec) of cells, incubated on a dish with cell wall digesting enzymes in a hypotonic buffer containing 0.5 M sorbitol. Left panel: cells in exponential growth, right panel: CF cells on starvation day 6. Scale bar: 5µm.

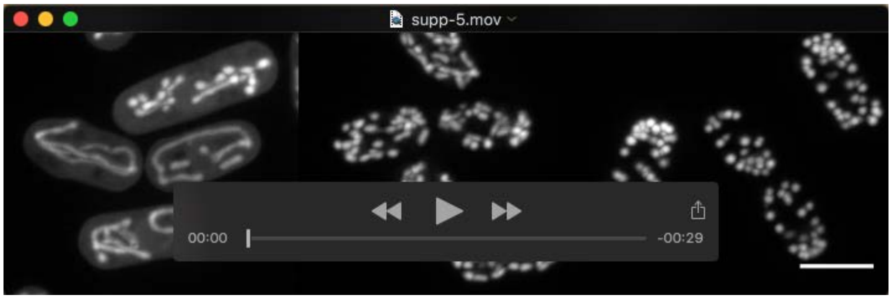

Movie 5 - Mitochondria dynamics during starvation

Movies (2frames/3sec) of mitochondria visualized by maximum intensity projections of cox4-GFP expressing cells during starvation. Panels show from left to right cells in exponential growth, cells on starvation day 3 and cells on starvation day 6. Scale bar: 5µm.

Journal of Cell Science • Supplementary information

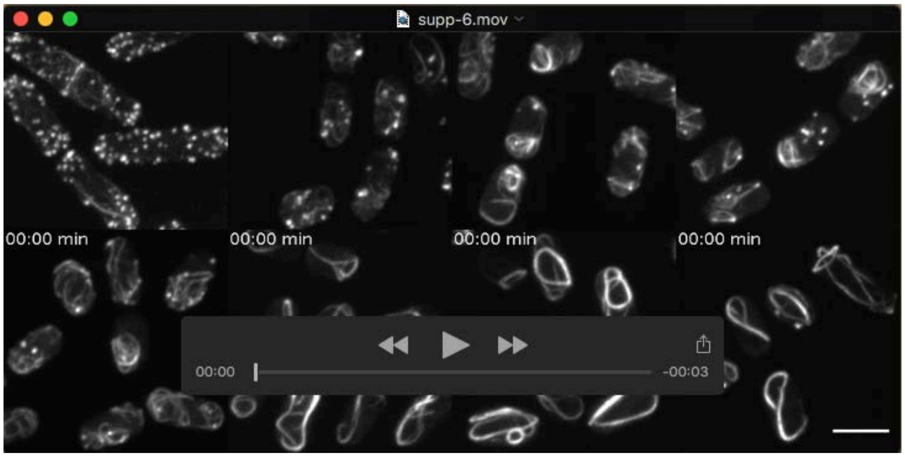

Movie 6 - F-actin organization and dynamics in starvation

Movies (1frame/5sec) visualizing F-actin dynamics based on maximum intensity projections of Lifeact-GFP expressing cells. Upper panels show from left to right, exponentially growing cells and cells at starvation days 2, 3 and 4 respectively. The lowers panels show cells at starvation days 5, 6, 7 and 8. Scale bar: 5μm.

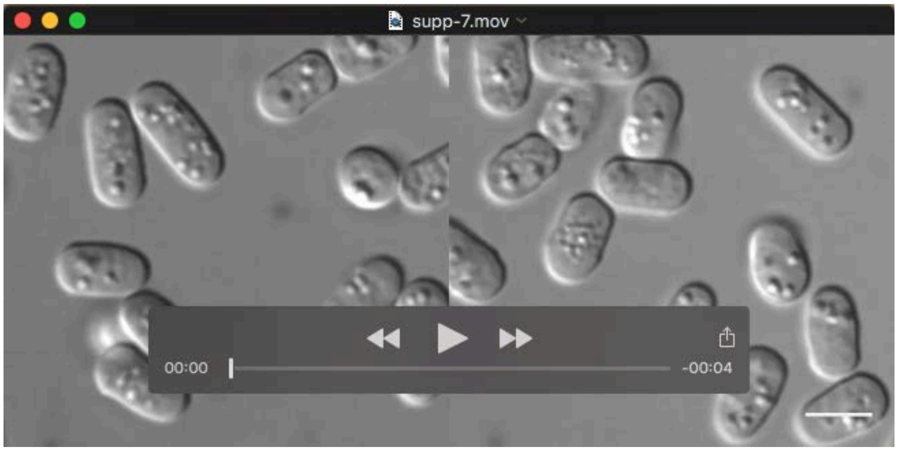

Movie 7 - CF in F-actin depleted cells

25sec DIC movies (4frames/sec) of wild type cells at starvation day 6 incubated from starvation day 3 onwards with DMSO (control, left panel) or LatB. Scale bar: 5μm.

Journal of Cell Science • Supplementary information

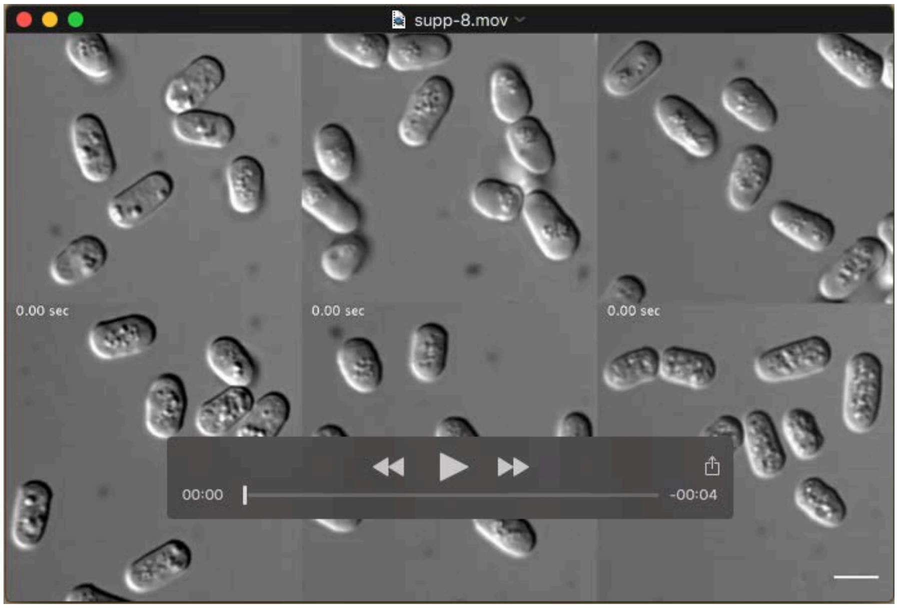

Movie 8 - Interference with autophagy delays CF

25sec DIC movies (4frames/sec) of, from left to right, wild type, *atg1*Δ and *atg8*Δ cells at starvation days 6 (upper panels) and 9 (lower panels). Scale bar: 5µm.

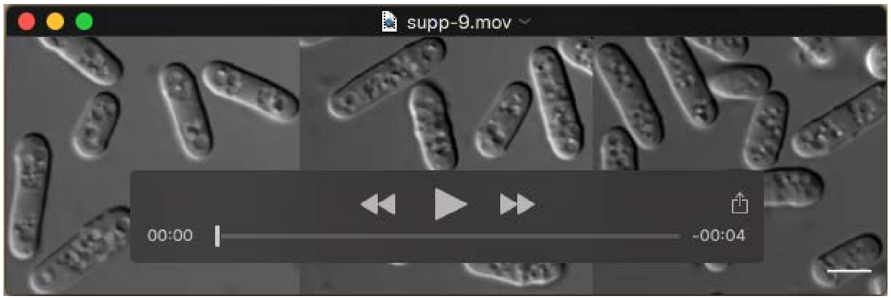

Movie 9 - LD motion in cells suffering of acute energy depletion

Left to right: 25sec DIC movies (4frames/sec) of AGD cells and DED cells with 2h and 0.5h drug treatment respectively. Scale bar: 5µm.

Journal of Cell Science • Supplementary information

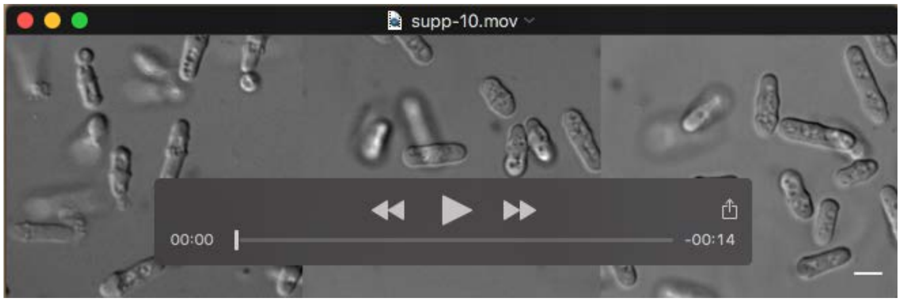

Movie 10 - Protoplasting of cells in hypotonic conditions

DIC movies (1frame/10sec) of cells, incubated on a dish with cell wall digesting enzymes in 0.5M sorbitol containing buffer. Panels show from left to right, AGD cells and DED cells with 2h and 0.5h drug treatment respectively. Scale bar: 5µm.

Journal of Cell Science • Supplementary information
